# A collaborative and near-comprehensive North Pacific humpback whale photo-ID dataset

**DOI:** 10.1038/s41598-023-36928-1

**Published:** 2023-06-23

**Authors:** Ted Cheeseman, Ken Southerland, Jo Marie Acebes, Katherina Audley, Jay Barlow, Lars Bejder, Caitlin Birdsall, Amanda L. Bradford, Josie K. Byington, John Calambokidis, Rachel Cartwright, Jen Cedarleaf, Andrea Jacqueline García Chavez, Jens J. Currie, Joëlle De Weerdt, Nicole Doe, Thomas Doniol-Valcroze, Karina Dracott, Olga Filatova, Rachel Finn, Kiirsten Flynn, John K. B. Ford, Astrid Frisch-Jordán, Christine M. Gabriele, Beth Goodwin, Craig Hayslip, Jackie Hildering, Marie C. Hill, Jeff K. Jacobsen, M. Esther Jiménez-López, Meagan Jones, Nozomi Kobayashi, Edward Lyman, Mark Malleson, Evgeny Mamaev, Pamela Martínez Loustalot, Annie Masterman, Craig Matkin, Christie J. McMillan, Jeff E. Moore, John R. Moran, Janet L. Neilson, Hayley Newell, Haruna Okabe, Marilia Olio, Adam A. Pack, Daniel M. Palacios, Heidi C. Pearson, Ester Quintana-Rizzo, Raul Fernando Ramírez Barragán, Nicola Ransome, Hiram Rosales-Nanduca, Fred Sharpe, Tasli Shaw, Stephanie H. Stack, Iain Staniland, Jan Straley, Andrew Szabo, Suzie Teerlink, Olga Titova, Jorge Urban R., Martin van Aswegen, Marcel Vinicius de Morais, Olga von Ziegesar, Briana Witteveen, Janie Wray, Kymberly M. Yano, Denny Zwiefelhofer, Phil Clapham

**Affiliations:** 1 Happywhale, Santa Cruz, California USA; 2 Southern Cross University, Lismore, NSW Australia; 3 BALYENA.ORG, Jagna, Philippines; 4 Whales of Guerrero, Barra de Potosí, Mexico; 5 NOAA Southwest Fisheries Science Center, San Diego, California USA; 6 Hawaiʻi Institute of Marine Biology, University of Hawaiʻi at Manoa, Kaneohe, Hawaiʻi USA; 7 Marine Education and Research Society, Port McNeill, British Columbia Canada; 8 Ocean Wise, Vancouver, British Columbia Canada; 9 NOAA Fisheries Pacific Islands Fisheries Science Center, Honolulu, Hawaiʻi USA; 10 Pacific Wildlife Foundation, Port Moody, British Columbia Canada; 11 Cascadia Research Collective, Olympia, Washington USA; 12 Keiki Kohola Project, Delray Beach, Hawaiʻi USA; 13 University of Alaska Southeast, Juneau, Alaska USA; 14 Pacific Whale Foundation, Maui, Hawaiʻi USA; 15 Association ELI-S, Gujan-Mestras, France; 16 Fisheries and Oceans Canada, Nanaimo, British Columbia Canada; 17 North Coast Cetacean Society, Hartley Bay, British Columbia Canada; 18 University of Southern Denmark, Odense, Denmark; 19 NOAA Hawaiian Islands Humpback Whale National Marine Sanctuary, Kihei, Maui, Hawaii USA; 20 Ecología y Conservación de Ballenas, Puerto Vallarta, Mexico; 21 Glacier Bay National Park and Preserve, Gustavus, Alaska USA; 22 Hawaiʻi Marine Mammal Consortium, Kamuela, Hawaiʻi USA; 23 Eye of the Whale Marine Mammal Research, Kamuela, Hawaiʻi USA; 24 Marine Mammal Institute, Oregon State University, Newport, Oregon USA; 25 Cooperative Institution of Marine and Atmospheric Research, Research Corporation of the University of Hawaiʻi, Honolulu, Hawaiʻi USA; 26 VE Enterprises, Arcata, California USA; 27 Departamento Académico de Ingeniería en Pesquerías, Universidad Autónoma de Baja California Sur, La Paz, Baja California Sur México; 28 Whale Trust, Puunene, Hawaiʻi USA; 29 Okinawa Churashima Foundation, Kunigami-gun, Japan; 30 Humpback Whales of the Salish Sea, Duncan, British Columbia Canada; 31 Commander Islands National Park, Kamchatka Krai, Russian Federation; 32 Universidad Autónoma de Baja California Sur, La Paz, Mexico; 33 NOAA Alaska Fisheries Science Center, Juneau, Alaska USA; 34 North Gulf Oceanic Society, Homer, Alaska USA; 35 University of Hawaiʻi at Hilo, Hilo, Hawaiʻi USA; 36 The Dolphin Institute, Hilo, Hawaiʻi USA; 37 Department of Fisheries, Wildlife, and Conservation Sciences, Oregon State University, Newport, Oregon USA; 38 Emmanuel College, Boston, Massachusetts USA; 39 Murdoch University, Perth, WA Australia; 40 Alaska Whale Foundation, Petersburg, Alaska USA; 41 International Whaling Commission, Impington, UK; 42 NOAA Fisheries Alaska Regional Office, Juneau, Alaska USA; 43 Severtsov Institute of Ecology and Evolution, Moscow, Russian Federation; 44 Winged Whale Research, Homer, Alaska USA; 45 University of Alaska Fairbanks, Fairbanks, Alaska USA; 46 Seastar Scientific, Vashon Island, Washington USA

**Keywords:** Software, Marine biology, Population dynamics, Conservation biology

## Abstract

We present an ocean-basin-scale dataset that includes tail fluke photographic identification (photo-ID) and encounter data for most living individual humpback whales (*Megaptera novaeangliae*) in the North Pacific Ocean. The dataset was built through a broad collaboration combining 39 separate curated photo-ID catalogs, supplemented with community science data. Data from throughout the North Pacific were aggregated into 13 regions, including six breeding regions, six feeding regions, and one migratory corridor. All images were compared with minimal pre-processing using a recently developed image recognition algorithm based on machine learning through artificial intelligence; this system is capable of rapidly detecting matches between individuals with an estimated 97–99% accuracy. For the 2001–2021 study period, a total of 27,956 unique individuals were documented in 157,350 encounters. Each individual was encountered, on average, in 5.6 sampling periods (i.e., breeding and feeding seasons), with an annual average of 87% of whales encountered in more than one season. The combined dataset and image recognition tool represents a living and accessible resource for collaborative, basin-wide studies of a keystone marine mammal in a time of rapid ecological change.

## Introduction

Understanding the population ecology of a species is crucial for conservation management, but studies of most migratory marine species are compromised by data deficiency. Individual identification through techniques such as photographic identification (photo-ID), radio telemetry, and genetic sequencing allow researchers to track individual animals over time. This enables population modeling, revealing movement patterns, social interactions, and reproductive success rates. Photo-ID, in which a photograph of persistently identifiable features of an individual is recorded together with its date and location, offers an efficient and non-invasive data collection method^1^. For long-lived migratory species, effective population studies require extensive data collection, including the additional challenges of collaboration across regional and international boundaries.

The humpback whale, *Megaptera novaeangliae*, is a globally distributed baleen whale species with a complex population structure and major ecosystem impacts^2–4^. Individuals engage in extensive seasonal migrations between high-latitude feeding areas during the spring, summer, and fall, and low-latitude tropical waters to mate and calve in winter and spring^2,4–8^. The long-distance migrations undertaken by humpback whales expose populations to diverse management regimes, anthropogenic risks, and ecological conditions^9^. For example, a very large marine heatwave in the North Pacific from late 2013–2016^10–12^ caused major negative impacts on humpback whale food resource availability. This resulted in sharp declines in abundance, survival, and reproductive success of humpback whales in Hawaiʻi and Southeast Alaska^13–17^. In a changing oceanic ecosystem, a cost-effective and non-invasive technique that repeatedly samples most living individuals can offer valuable insights into the status of the species and its ecosystem.

Humpback whale populations worldwide were severely depleted by extensive commercial whaling until late in the twentieth century. This species was listed under the U.S. Endangered Species Act (ESA) in 1970 due to an estimated 31,785 killed in the North Pacific from 1900 to 1979^18–20^. Following a global ban on humpback whale catches by the International Whaling Commission in 1966, and the cessation of Soviet illegal whaling in the following decade^19^, the humpback whale population has grown. Two studies have evaluated the abundance of humpback whales in the full North Pacific: first in the 1990s^4^, then a study entitled Structure of Populations, Levels of Abundance and Status of Humpback Whales (SPLASH) conducted from 2004 to 2006^8^. These studies estimated total North Pacific humpback whale abundance at 21,063 individuals in 2006, with an annual growth rate of 8.1% between the two study periods^21^. A major portion of SPLASH relied on the identification and resighting of individual humpback whales through photo-ID. This method involved trained observers visually matching photographs of the ventral side of each whale’s tail (flukes) based on unique white and black pigmentation patterns, together with unique fluke trailing edge contours^22,23^. SPLASH documented 7,640 individual humpback whales in 18,469 unique encounters (defined as a single sighting of a unique individual supported by a referenced photo-ID image, recorded on a specific day at a specific location); these encounters occurred across all known breeding and feeding areas. SPLASH reinforced the value of broad-scale data sharing and collaboration, and exposed gaps in knowledge of humpback whale status in the North Pacific.

In 2016, NOAA Fisheries, pursuant to the ESA, defined 14 humpback Distinct Population Segments (DPSs) globally using photo-ID data and other lines of evidence^24^. DPS designations are based on theoretically discrete breeding areas where many whales show long-term site fidelity^25^. In feeding areas, whales also show high site fidelity and arguably face greater biological and anthropogenic stressors^26^. Four DPSs occur in the North Pacific, with breeding occurring in waters off Central America, Mexico, Hawaiʻi, and the Western North Pacific (Mariana Islands, the Philippines, and Japan). Based on varying rates of recovery, the Central America and Western North Pacific DPS remain listed as Endangered (s), the Mexico DPS is considered Threatened, and the Hawaiʻi DPS has been deemed to not warrant listing^27^. Ironically, removal of the Hawaiʻi DPS's endangered status by the US coincided with the 2013–2016 marine heatwave that negatively affected population health^13–16,28^.

Individual photo-ID data have advanced the understanding of humpback whale behavior, ecology and conservation issues based on many regional study efforts^13,14,25,29–41^. However, after SPLASH ended in 2006, local and regional photo-ID datasets were seldom integrated with one another. This was in part due to prohibitively time-intensive visual matching of individual ID fluke photos in ever-growing catalogs. The current study established the North Pacific Humpback Whale Photo-ID (NPPID) collaboration. The goal of this collaboration was to integrate and advance knowledge of humpback whale population structure and migratory movement in the North Pacific through creation of a shared repository of resighting data for individual whales across the full study region. A central objective of the effort was to implement a collaborative framework to facilitate data availability, access, and readiness. Given the large amount of data involved and the difficulty of obtaining long-term funding, to be successful the system needed to drive the incremental cost of acquisition of each successive datapoint to near zero. Such a system required effective technology and web-based data management to submit, quality-control, identify, and curate encounter data for a growing set of known individual whales. The NPPID was built on newly established automated fluke photo-ID matching technology. This technology achieves a measured 97–99% accuracy with good- to high-quality images and is orders of magnitude faster than manual visual matching^42^. However, a system is not technology alone; the system needed to sustainably nurture positive collaboration practices to bring together the many contributors responsible for tens of thousands of whale encounters per year. Therefore, the NPPID was developed as a shared effort utilizing the user-friendly and interactive web-based platform, www.Happywhale.com (Happywhale). Here we describe the process of building this ocean-basin-wide ongoing photo-ID collaboration involving 43 research groups and thousands of public contributors (also known as "community scientists" or "citizen scientists"). This approach has enabled rapid feedback for population and longitudinal studies of humpback whales across the North Pacific. The process and framework described here have broader practical relevance for navigating the use of complex multi-contributor datasets.

## Materials and methods

### The North Pacific humpback whale Photo-ID (NPPID) collaboration

This effort began in 2018 as a data-sharing initiative to revive the collaboration established with the 2004–2006 SPLASH study^8^, supplemented by photo-ID images from community scientists. We built upon the SPLASH dataset, study methodology, and collaboration, but did not have a budget for data acquisition or fieldwork. All SPLASH collaborators known to be active in North Pacific humpback whale studies were invited to join, along with all known newer regional researchers and organizations. Data collection relied on existing archives and ongoing field efforts by the individual collaborators. All dedicated data collection by study collaborators was carried out in accordance with permitting requirements of respective authorities (permit details are listed in acknowledgements). Data collection from community scientists was sourced primarily from whale watch companies operating under regulations and guidelines of respective national, regional, and local authorities. A primary incentive for participation in the NPPID collaboration was the promise of novel and fully automated image-recognition technology^42^ that effectively eliminated the cumbersome, time-intensive visual matching process from photo-ID data management.

Through a memorandum of agreement (MOA, Supplementary Material I), all research organizations in the NPPID committed to sharing photo-ID images and associated supporting data for every available encounter, with a focus on a 2001–2021 study period. The specific research aim was to further knowledge of population structure and migratory movement via photographic mark-recapture population model development ^e.g.^^21,43^. The broader aim was to create an ongoing, living dataset for continued population monitoring. Under the MOA terms, each data contributor chose whether their data were publicly visible via Happywhale or visible only to collaborators who had signed the MOA. The MOA limited data use to a defined set of publications about population status and migratory patterns; any additional use required agreement from all collaborators. The infrastructure, compiled data, and collaborator connections will remain after the period of the current MOA. Therefore, its use needs to be addressed with further agreement among collaborators if the dataset is going to be an ongoing, living entity.

### Data integration and quality control

Humpback whale encounter data were delivered to Happywhale data managers from collaborators in a wide range of states of reconciliation, from unmatched original scans and digital photos to fully edited sets of images (i.e., exposure adjusted as needed and cropped tightly around flukes), with IDs assigned to each individual whale. The minimum data required for each encounter were: date, location, and photo-ID image or confirmed individual ID. All encounters of each whale were preserved, and all available supporting attribute data were maintained with the encounter; this could include filename, date, time, location, individual ID from the collaborator’s naming/numbering system, observer names, vessel name, observed whale sex, age class, health, behavior, group composition and any further observations. Because the state of every dataset varied at the time of delivery, all data were managed through the following standard series of steps:

1. **Image management and matching**: Images were quality-controlled through cropping tightly around the flukes and, if necessary, exposure adjustment to facilitate algorithmic ID followed by visual ID confirmation. All images were quality-scored on a 0–5 scale as described in a previous study^42^, where 0 represented photos in which no photo-ID features were visible, and 1–5 represented very poor to excellent quality photos, respectively. All photo-ID images were matched to a progressively growing set of known whales via an automated image recognition system^42^. Every match proposed by the system was manually confirmed by a trained observer. All matches that could be visually confirmed by a trained observer were maintained regardless of image quality. A previous study established that 97–99% of potential matches are found by this method for good- to high-quality images^42^.
2. **Supporting attribute data curation**: Given the diversity of supporting data formats received, standardization was necessary for dataset management. Locations were categorized as general (confident of location within 200 km [km]), approximate (confident of location within 20 km), or precise (confident of location within 2 km). Within the precise location category, location data source was categorized as: (a) camera GPS embedded into the image, (b) synchronous GPS track, (c) pinpoint recorded from a GPS unit, (d) pinpoint recorded via a mobile app, or (e) manually transcribed record. For encounters without a known date, an approximate date to month, season or year was assigned as information allowed, with date precision noted in encounter attributes. Encounters without a date known at least to year or location known confidently within 200 km were excluded. Descriptive observational data and contextual information such as whale sex, age class, behavior, mother/calf relationships or group composition, and scarring (e.g., from entanglement, ship strike, killer whales) were recorded with each encounter when available and without standardization. Data quality was reviewed on import, with an opportunity for review by both data managers and data contributors before entry into a relational database.
3. **Efficiency with large datasets**: To increase efficiency for collaborators with large, well-curated datasets, some encounters were accepted with an individual ID name/number and supporting date, location, and attribute data, without a photo-ID image linked to every encounter. These encounters were linked to known individuals represented in one or more catalog photos.

### Many-to-one reference catalogs

All images were automatically matched against all individual humpback whales known at the time of each respective dataset integration. Across the NPPID study area, 39 separate catalog systems were received that had collaborator-specific individual IDs (Table 1). These ID naming systems were accommodated into a many-to-one ID structure so that any individual could be tracked via any of the multiple catalog IDs assigned to them.

**Table 1:** Humpback whale photo-ID reference catalog naming systems integrated in this study.

Reference catalog	Code	Individuals	Number of 2001–2021 encounters	Regions
Alaska Whale Foundation	AWF	780	10,508	Southeast Alaska
BALYENA.ORG^75^	BALYENA	228	1193	Philippines
Bree Witteveen Alaska Catalog	BREE	1906	7959	Alaska Peninsula and Gulf of Alaska
MML Cetacean Assessment and Ecology Program	CAEP	412	3889	Pelagic North Pacific
Association ELI-S	CCN	124	1713	Nicaragua
Canadian Pacific Humpback Collaboration	CPHC	1197	14,569	British Columbia
Cascadia Research Collective^4,76–79^	CRC	7017	66,967	British Columbia to Central America, mostly US West Coast
Clayoquot and Barkley Sound	CS	530	6094	Central British Columbia
DFO Canada	DFO	1982	26,494	British Columbia
Eye of the Whale	EOTW	172	1589	Gulf of Alaska and Hawaiʻi
ECOBAC FIBB Catalog	FIBB	2818	27,130	Mexico
Glacier Bay National Park & Preserve	GBNP	72	732	Glacier Bay and Icy Strait, Southeast Alaska
Gulf Watch Alaska	GWAK	361	2216	Kenai Fjords and Prince William Sound, Gulf of Alaska
Hawaiian Islands Humpback Whale National Marine Sanctuary	HIHWNMS	1051	7368	Hawai’i
Happywhale^42^	HW	11,851	78,070	Pan-Pacific
Juneau Flukes	JUNEAU	19	1180	Juneau, Southeast Alaska
Kachemak Bay Whales	KBAY	391	2188	Kachemak Bay, Gulf of Alaska
Keiki Kohola Project	KKP	33	198	Hawai’i
Marine Education and Research Society	MERS	308	7288	Central and southern British Columbia
Humpbacks of the Salish Sea (HWSS)	HWSS	483	7853	Salish Sea
Marine Mammals of Oaxaca	MMO	65	781	Oaxaca, Mexico
North Coast Cetacean Society	NCCS	329	5169	Northern British Columbia
North Coast Cetacean Research Initiative	NCCRI	188	1860	Northern British Columbia
North Gulf Oceanic Society	NGOS	39	396	Gulf of Alaska
Okinawa Churashima Foundation^75^	OCF	1732	6322	Okinawa, Japan
Oregon State University Marine Mammal Institute Whale Habitat, Ecology, and Telemetry Laboratory^80^	OSUWTG	1242	14,765	Central and eastern Pacific
Pacific Islands Fisheries Science Center, NOAA Fisheries^35^	PIFSC	92	245	Mariana Islands and Northwestern Hawaiian
Pacific Whale Foundation	PWF	5022	33,346	Hawai’i-focused, pan-Pacific
Prince William Sound Catalog	PWS	184	1570	Prince William Sound, Alaska
Russian Cetacean Habitat Project^75,81^	RCHP	2028	6545	Russia
Sayulita Humpback Whale	SAYU	145	1376	Sayulita, Mexico
Southeast Alaska Humpback Whale Catalog	SEAK	2571	34,284	Southeast Alaska
SPLASH Project^8,21^	SPLASH	7838	64,144	Pan-Pacific
The Dolphin Institute^25^	TDI	1055	5970	Hawai’i and Southeast Alaska
The Marine Mammal Center	TMMC	67	1827	San Francisco Bay, California
Programa de Investigación de Mamiferos Marinos, Universidad Autónoma de Baja California Sur	UABCS	601	3950	Baja California Sur and Revillagigedo Islands, Mexico
Universidad Nacional Autónoma de Mexico	UNAM	1053	9045	Mexico
Whales of Guerrero	WGRP	329	5242	Guerrero, Mexico
Whale Trust	WTM	1994	15,708	Hawai’i

A single unified dataset allows cross-referencing of all known IDs for each individual. Some collaborating research groups share naming systems; all IDs are accounted for only once in the table below.

### Reconciliation of duplicate IDs

Every image was matched within and among all collaborator catalogs. One individual ID per catalog was allowed. Thus, if individuals were found with duplicate IDs due to false negatives (where a previously undetected match of one whale with two or more separate IDs within a collaborator catalog was found), the contributor chose the persisting ID (typically the lowest of a sequential ID series). Each duplicate ID was noted in the attributes for the individual whale. Newly detected (i.e., unmatched) individuals were added to the continually growing reference set, with the collaborator ID, if available, or with a newly assigned Happywhale catalog ID. False positives (where two different whales were combined into one individual record) were minimized through trained observer review of every match.

### Community science data contributions

Opportunistic images submitted through Happywhale were also matched against all known whales, supplementing the research collaboration with community science-sourced encounter data. The same image and data quality control standards were applied as described above. All community science data contributors implicitly acknowledged their choice of data usage rights during the submission process and had the option of changing usage rights settings among established levels of Creative Commons usage rights (https://en.wikipedia.org/wiki/Creative_Commons_license). Unlike research collaborators participating under the terms of the MOA, public contributors did not have the option of restricting public visibility. Public contributors had access to an encounter comment system whereby suspected data errors and outliers could be brought to the attention of data managers, creating a feedback loop for review and error detection.

### Information system structure and development

**Figure 1:**
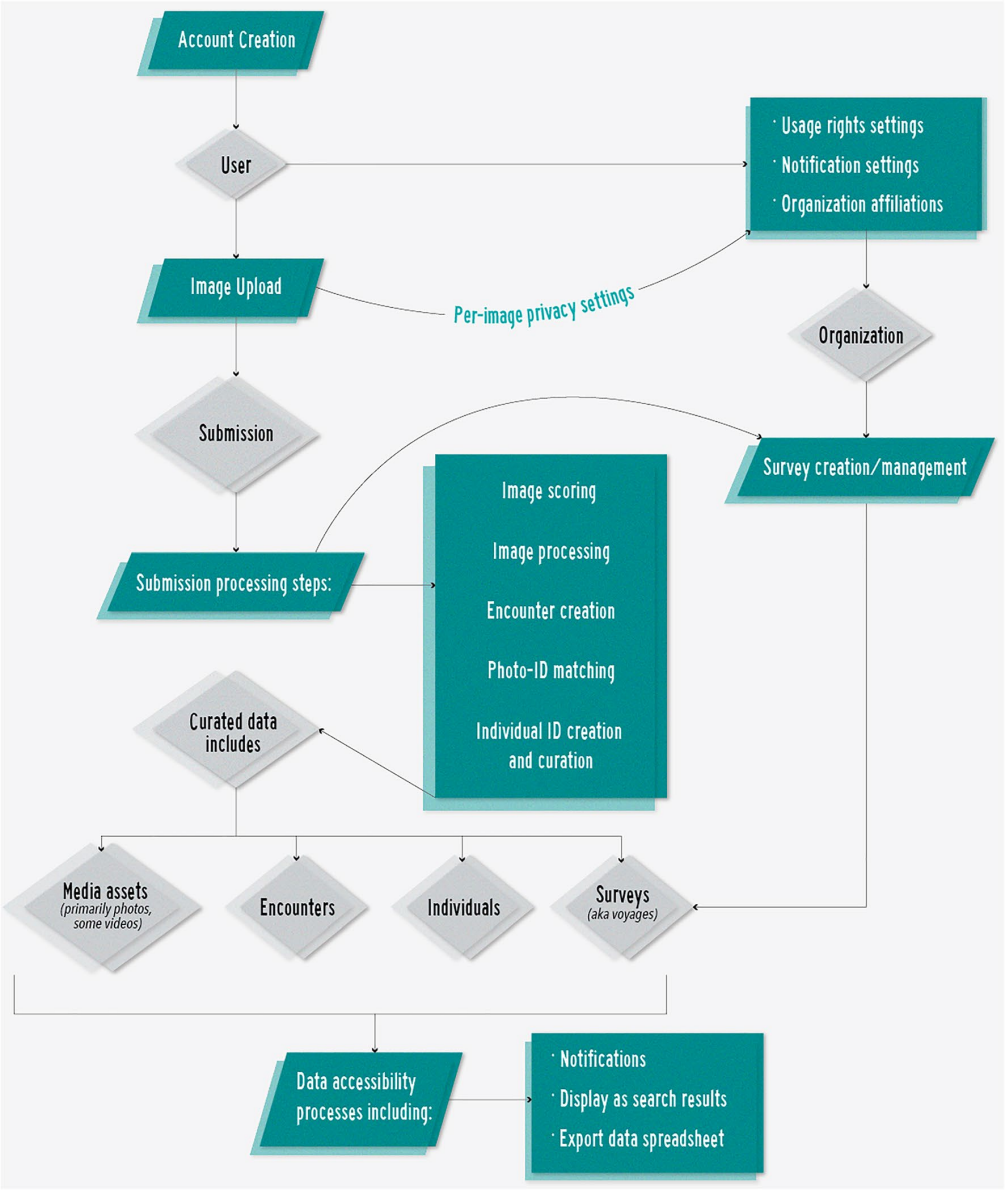
Happywhale simplified data process and elements. Processes (in teal) of user creation, media upload, submission processing and accessibility lead to creation of elements (in gray) of users, organizations, submissions, and curated data.

The NPPID data management system integrated a workflow of image processing, individual identification, and recording and curating encounter and individual attribute information. Data were structured through units of contributors (i.e., “users”), images, encounters, individual humpback whales, and surveys (i.e., “voyages”), linked by a series of workflow processes (Fig. 1). The cloud-based information architecture was composed of a dedicated server for the Java Spring application using a PostgreSQL database populated with Darwin Core compatible fields^44^. Submitted binary media were stored in a Simple Storage Service (S3) system for global retrieval. The ID system used a combination of a Node server and a Python Flask app to run the PyTorch-based ID algorithm.

During the collaboration, ongoing system development brought enhanced functionality and sophistication to data management processes within the Happywhale.com web platform. In 2021, the automated image recognition system was rebuilt to deliver results in under 0.1 s per image. This efficiency reduced server load, which has accommodated direct access by collaborators to batch process photo-ID images directly via web and mobile app interfaces in the lab or field. Near-instantaneous access to image processing was adopted by many collaborators to facilitate more efficient and effective internal data management.

NPPID collaborators were invited to directly manage their data import process and ongoing curation, with training, feedback, and quality control oversight by system managers. Some collaborators used the system as a principal repository of their data while others maintained their own separate data management systems during the study. As import and management tools developed in a constantly evolving system, collaborators were increasingly enabled and encouraged to manage their own data.

### System use, public outreach, and data accessibility

The FAIR Principles (Findable, Accessible, Interoperable, and Reusable) for scientific data^45^ guided system design. Public awareness of the opportunity to contribute to whale conservation science was spread through word of mouth, social media, and documentary films. The primary focus of outreach was to seek and reach naturalists, whale watch guides and enthusiasts already familiar with the concept of marine mammal photo-ID, and equipped with camera gear sufficient to create quality images. Community scientists and NPPID collaborators were promised they would be rewarded with knowledge. This was accomplished through a notification system with alerts to novel developments regarding individuals they had encountered (e.g., initial identification typically within a few days of submission, discovery of duplicate IDs, and ongoing resightings). Would-be contributors were directed to Happywhale with little guidance beyond a request for humpback whale photo-ID photos from any date and location, as long as the contributor could confirm the date and location. The data upload process sought to balance ease of access with rigor for data quality, with data validation dependent upon the image management process.

**Figure 2:**
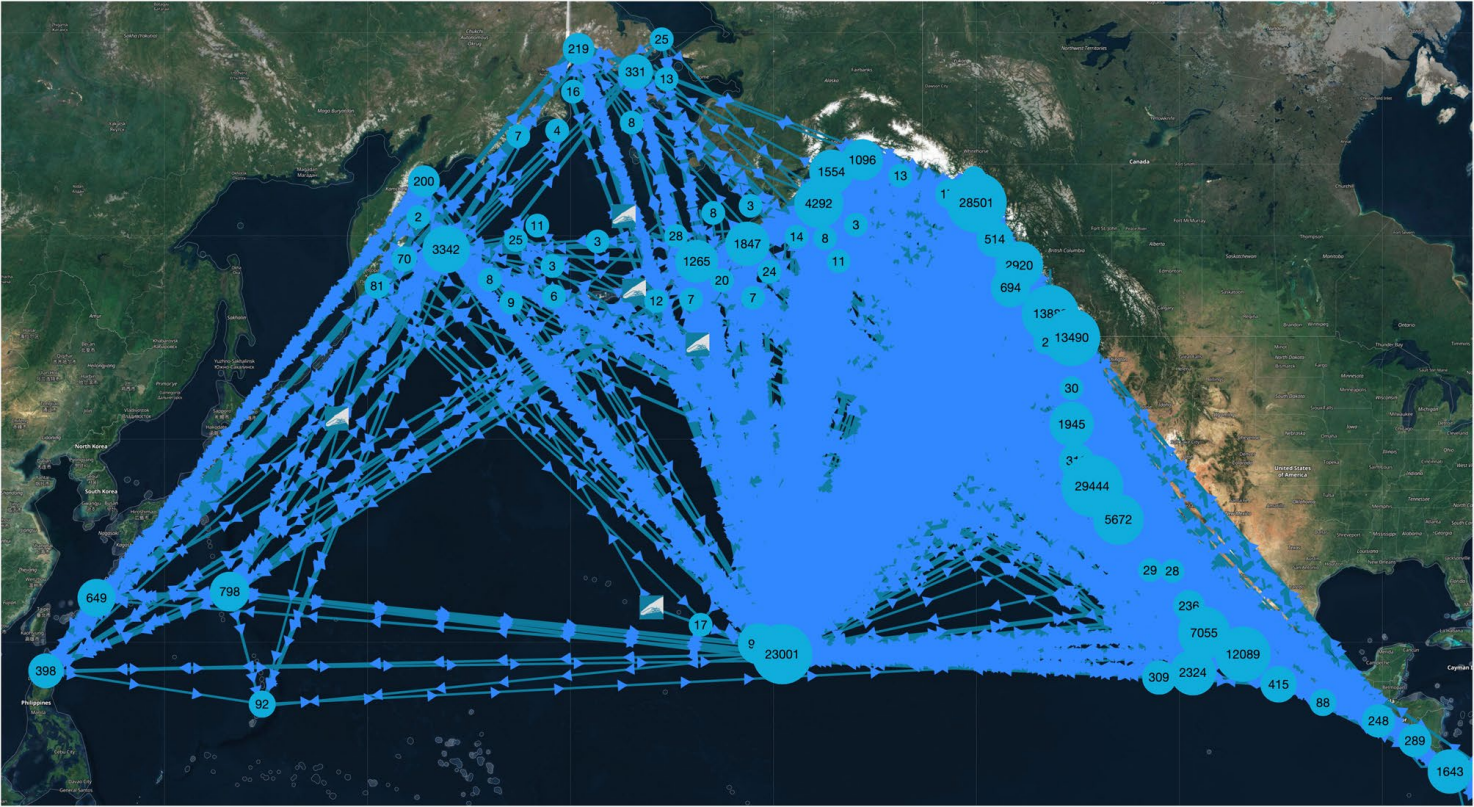
All North Pacific humpback whale encounters and migratory connections as viewable in Happywhale map view for all data collected through August 2022. Numbers in blue circles are counts of individual encounters aggregated by area, while the humpback whale icon represents a single encounter. Blue lines and arrows represent migratory connections of whales sighted in more than one location, not actual travel paths. Map created using Happywhale, built on a basemap reproduced with permission from Maptiler (www.maptiler.com) and OpenStreetMap (www.openstreetmap.org).

Data are searchable and accessible in ‘map view’ (Fig. 2) and ‘list view’ formats via Happywhale. Users can expand a search from a set of encounters (for example, all encounters contributed by one user or all encounters in a geographic area in a defined time period) to all sightings globally of individuals within the found set. This allows quick visual exploration of migratory connections for any set of whales. For collaborators, data are available for export into a standard comma-separated value (CSV) format, translatable to downstream analytical and research processes in GIS or statistical software.

### Analytics—Documenting detection probability

**Figure 3:**
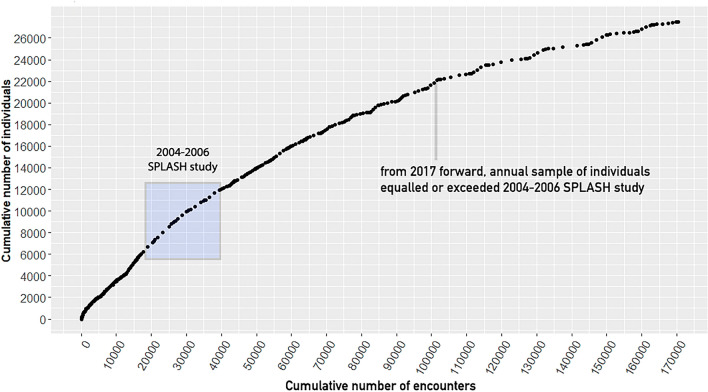
Discovery curve of cumulative number of North Pacific individual humpback whales versus cumulative number of encounters for all data collected through August 2022. Each dot represents one month of effort. The 2004 through 2006 SPLASH study resulted in a large increase in known whales during the study’s three years. From 2017 forward, at 101,000 cumulative encounters, the annual number of individuals identified matched or exceeded SPLASH annual sample sizes, yet the cumulative number of individuals increased by an average of only 5% annually, compared to 21% during SPLASH.

**Figure 4:**
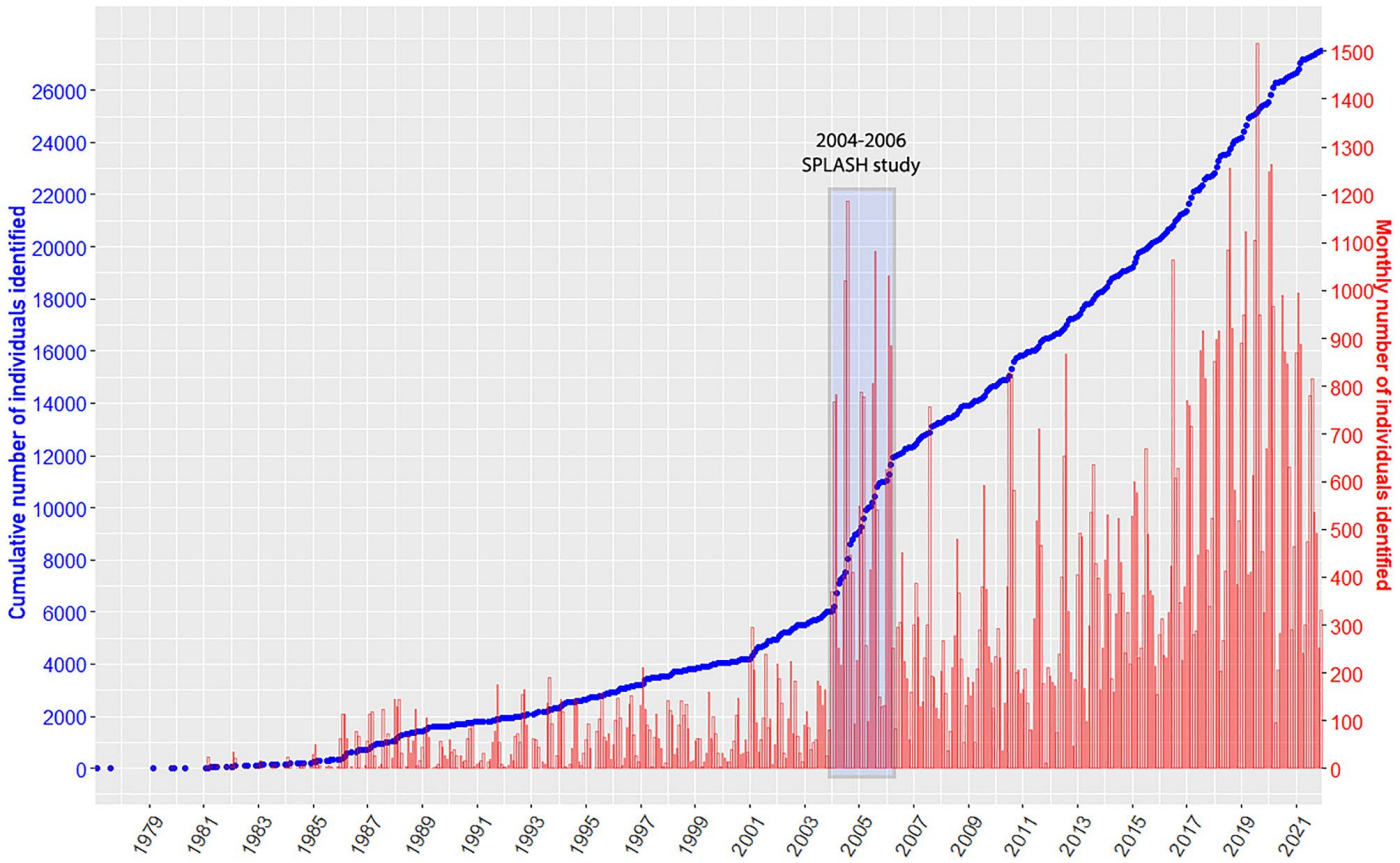
Cumulative individual identifications over time for the number of uniquely identified individual humpback whales documented in the North Pacific for all available photo-ID records collected through August 2022. Dates refer to the time when whales were photographed. Field effort during the 2004–2006 SPLASH study, highlighted in light blue, resulted in a steep increase in the total number of individuals identified.

The 2004–2006 SPLASH project actively developed collaborations and supported field efforts in all known (at the time) North Pacific humpback whale breeding and feeding areas, in pursuit of comprehensively representative sample sizes^8^. In contrast, the NPPID project relied on contributions from existing datasets, ongoing field efforts, and community science image contributions. With successive integration of datasets, detection probabilities progressively increased, leading to a predominance of resightings (documenting an individual multiple times) and fewer new whales added to the comprehensive catalog. This caused a shift in methodology from predominantly cataloging new whales to confirming matches of known whales. To understand the proportion of the populations sampled in our growing known dataset, we plotted a discovery curve of new versus total identified individuals (Fig. 3), and a modified discovery curve of individuals identified over time (Fig. 4), in order to describe effort over the course of the history of the dataset.

## Results

**Figure 5:**
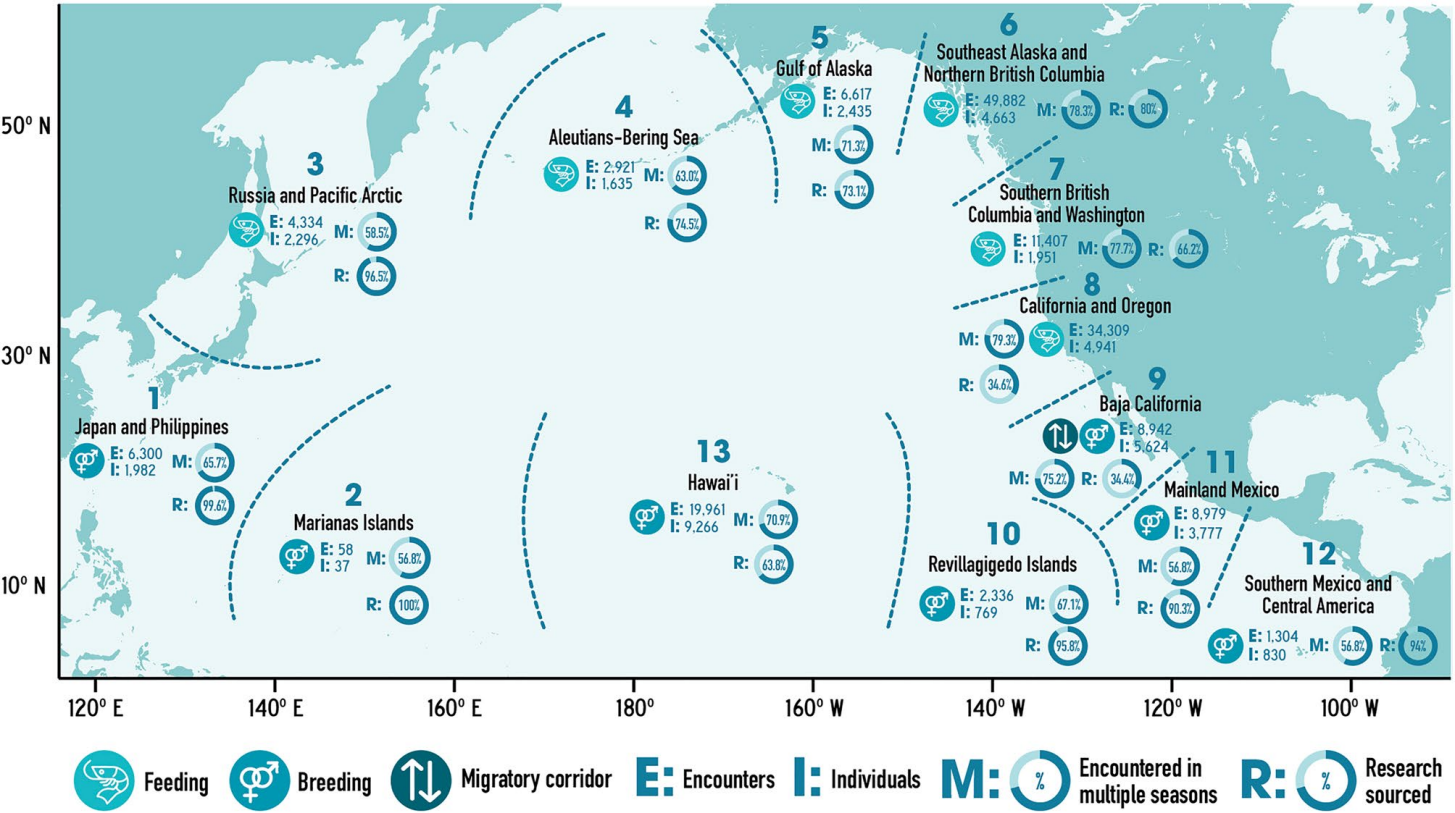
Humpback whale photo-ID data collections by region across the North Pacific Ocean. Region boundaries are indicated by dashed lines, with numbering that corresponds with Table 3. Data for each region includes: a symbol indicating feeding, breeding, or migratory corridor, E: a count of all encounters (trimmed to one encounter per individual per season) documented in each region, I: a total count of individuals documented in each region, M: the percentage of individuals encountered in more than one sampling season, and R: the percentage of data sourced from research collaborators versus community science. Map created with Adobe Illustrator 27.5 on an open source basemap from Freepik.com.

The NPPID collaboration involved 43 research organizations and included data from all nations around the North Pacific rim where humpback whales are known to regularly occur (Tables 1 and 2, Fig. 5). The complete NPPID collaboration ocean-basin dataset totaled 30,100 individual whales (February 1977 through August 2022 encompassing all available data). A total of 27,956 unique individuals were documented in 157,350 encounters during the 2001–2021 study period (Table 2, Fig. 2). Effort was variable over time: it was much higher in some areas relative to others, and skewed to the central and eastern North Pacific. However, data collection occurred in all known humpback whale breeding and feeding areas, with high rates of individual resighting throughout (Table 3, Fig. 5). Approximately two-thirds of encounters were represented by a single photo-ID image, while the remaining third contained additional supporting images (e.g., multiple views of the flukes, dorsal fin to fluke series and/or behavioral and anatomical images of the same individual). Naming/numbering protocols for 39 reference catalogs were combined into one unified set, with an average of 1.96 IDs per individual (range: 1–10). Most encounters (66%, documenting 24,049 individuals) were sourced from NPPID collaborators, with the remaining 34% submitted by community scientists (documenting 15,298 individuals); these are shown by region in Fig. 5. The community science-sourced component of the dataset was contributed by 3413 Happywhale users (Supplementary Material II). By volume, most community science-sourced images were contributed by whale watch tour naturalists, who consistently photographed and uploaded photo-ID images of every whale they were able to photograph. Most encounters (66%) were made publicly visible, with the remainder visible only to NPPID collaboration members (Tables 1 and 2 by region and research group). An additional 6318 humpback whale encounters (4% of total North Pacific encounters, primarily from public contributors), remained unidentified to individual due to poor image quality.

**Table 2:** Dataset detail by NPPID collaborating research organization for all data contributed.

NPPID collaborating organization	Identified encounters	Individuals	Resighting ratio	% Publicly visible
Alaska Whale Foundation	3887	1387	2.8	26
Association ELI-S	133	129	1.0	98
BALYENA.ORG	479	231	2.1	57
Cascadia Research Collective	44,310	7117	6.2	99
Commander Islands National Park	579	564	1.0	100
Ecologia y Conservación de Ballenas, A.C. ECOBAC	6892	2998	2.3	48
Eye of the Whale Marine Mammal Research	247	229	1.1	100
Department of Fisheries and Oceans Canada	4680	1669	2.8	37
Glacier Bay National Park & Preserve	10,753	649	16.6	6
Happywhale	5085	1762	2.9	100
Hawaiʻi Marine Mammal Consortium	2702	1894	1.4	18
Humpback Whales of the Salish Sea	1990	517	3.8	1
International Whaling Commission	168	156	1.1	100
Juneau Flukes	377	73	5.2	89
Marine Education and Research Society	17,400	541	32.2	2
Oregon State University Marine Mammal Institute Whale Telemetry Group	1789	1281	1.4	100
Murdoch University	3985	2198	1.8	96
Pacific Islands Fisheries Science Center, NOAA Fisheries	142	99	1.4	65
NOAA Fisheries Southwest Science Center	1265	971	1.3	100
NOAA Fisheries Science Center, Alaska	2052	1373	1.5	91
NOAA Hawaiʻian Islands Humpback Whale National Marine Sanctuary	2309	1940	1.2	39
North Coast Cetacean Research Initiative, Ocean Wise	1080	280	3.9	0
North Coast Cetacean Society	5562	453	12.3	9
North Gulf Oceanic Society	1315	639	2.1	100
Okinawa Churashima Foundation	5989	1735	3.5	10
Pacific Whale Foundation	9730	5065	1.9	100
Pacific Wildlife Foundation, Canada	755	494	1.5	100
Russian Cetacean Habitat Project	3692	2057	1.8	61
Simmons University/ Emmanuel College	108	99	1.1	0
The Dolphin Institute	3706	2407	1.5	27
The Keiki Kohola Project	350	311	1.1	88
Universidad Autónoma de Baja California Sur (PRIMMA-UABCS)	2518	2100	1.2	100
University of Alaska Fairbanks	4934	2186	2.3	98
University of Alaska Southeast	5200	1945	2.7	34
University of Hawaiʻi at Mānoa, Hawaiʻi Institute of Marine Biology	341	305	1.1	100
VE Enterprises	1257	699	1.8	98
Whale Trust	2984	2183	1.4	93
Whales of Guerrero	698	571	1.2	100
Winged Whale Research	477	218	2.2	100

The resighting ratio statistic reports the average number of encounters of each identified individual.

**Table 3:**
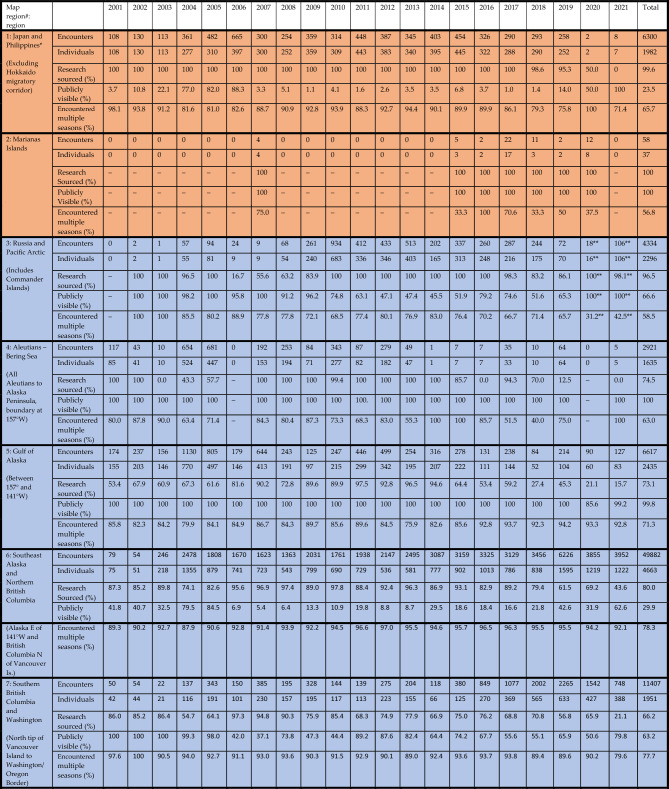

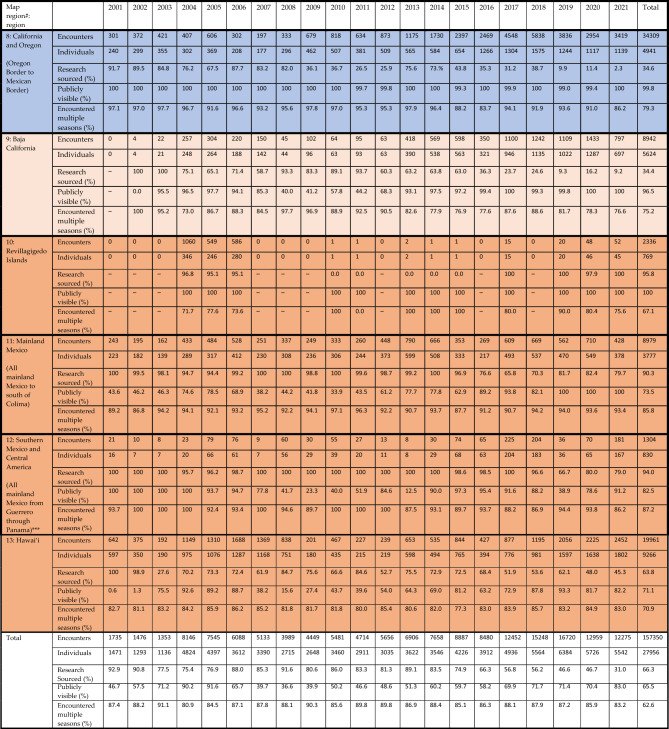
2001–2021 humpback whale dataset with sample size and characteristics presented by region and year.

Encounters: a count of the total number of photo-ID documented encounters of individuals, defined as one encounter per individual whale per day. Individuals: count of unique identified individuals. Research sourced: percent subset of encounters sourced from NPPID collaborators, while all remaining encounters were sourced from community science efforts. Public: percent subset of encounters visible to all users of Happywhale, while all remaining encounters are visible only to NPPID collaborators. Multiple seasons: a count of identified individuals documented in more than one season during the study, overall total for all years in final column. Seasons: north of 32.55°N = feeding regions (blue) data by calendar year; south of 32.55°N = migratory corridor (tan) and breeding regions (orange) data by breeding season, defined as 1 August through 31 July (i.e., a December 2015 encounter in Hawaiʻi is considered to be in the 2016 breeding season).

*Japan and Philippines data limited to one encounter per individual per year. **Russia 2020 and 2021 data limited to new individuals. ***Central America data from Nicaragua to Panama excludes Southern Hemisphere migrants encountered November through April of each year.

An annual average of 87% of individuals (84–92%) were documented in more than one season (Table 3, by region Fig. 5), averaging 5.6 seasons of encounters per individual. During the three-year SPLASH study, the cumulative number of individuals documented increased annually by an average of 21%. By contrast, from 2017 forward, with a comparable or greater number of individuals identified per year, cumulative individuals increased by an average of 5%, due to the documentation of a higher proportion of living individuals (Fig. 3). Data collection temporarily surged during the 2004–2006 SPLASH study, then increased gradually from 2007 and 2014 and more strongly from 2015 (Fig. 4).

Automated image recognition with manual review of each proposed match detected approximately 2,300 duplicate IDs (false negatives) within the 39 collaborator catalogs: these represent cases where the same whale was given multiple IDs within one catalog due to an undetected match (8% of total individuals). The range of false negatives across collaborator catalogs of greater than 100 individuals was 0.1–11%. In the SPLASH dataset of 7971 total individuals, 331 (4%) previously undetected false negatives were found. False positive errors, where two or more whales were confused as one individual, were far less likely than false negatives, prevented by manual review of each proposed match. False positives error rates were estimated to be below 0.1%. Over 5700 encounter comments were received through Happywhale's online comment fields from researchers and community scientists, in many cases alerting data managers to potential errors in date, location and/or whale identities.

## Discussion

The NPPID collaboration established a comprehensive, broad-scale, and rich dataset made possible by a rapid and rewarding feedback process connecting collaborator and community science data around the North Pacific Ocean basin. The NPPID collaboration is the first of its kind to develop a long-term individual ID database on this scale. This effort established a unique dataset foundation well-suited for humpback whale population modeling, as well as for any research benefitting from individual identification, such as longitudinal studies of individual health.

This study began during the development of fast and accurate automated image recognition for humpback whale flukes and demonstrated the scalability for the algorithm used. We could not initially predict how comprehensively we might document the populations of humpback whales across the NPPID study area. However, in a relatively short period the results exceeded expectations. As of August 2022, 56 months after the creation of this study, 30,100 individual North Pacific humpback whales had been documented. Some regions are now extremely well sampled. For example, in Southeast Alaska and northern British Columbia for 2011–2019, fewer than 6% of individuals encountered each year were unique (encountered in only one season) (Table 3, Fig. 5). The annual set of newly documented individuals includes recruitment of calves and juveniles, and a progressively smaller proportion of previously undocumented adults.

Data gaps exist, particularly in the western North Pacific, in remote feeding areas such as the Aleutian Islands, and in the Mexican offshore breeding area of the Revillagigedo Islands, where effort was far less than in most breeding, feeding and migratory corridor areas of the central and eastern Pacific. In the Northwestern Hawaiian Islands archipelago, recent acoustic-based surveys including those using wave-glider technology have revealed substantial singing and thus humpback whale abundance with relatively little fluke ID effort^46–48^. It remains to be determined if the majority of these whales use this region as a terminal breeding ground, or whether they mix during a breeding season with those whales in the main Hawaiian Islands. However, even in these least-sampled regions, over 50% of individuals were encountered in more than one season, in the same or in different regions. Thus, we believe that the great majority of individuals in all the North Pacific, including the less sampled regions, are documented in the NPPID dataset. By extensively resampling populations in breeding grounds, migratory corridors, and feeding areas, the impact of effort bias on population models can be reduced^21^. We believe applies to the NPPID dataset.

### Accessibility and user agreements

Data collection should not be an end unto itself, and sharing is a core tenet of good data management^49^. The Happywhale web platform was developed to make data accessible by design, aiming for a user experience that is both easy and rewarding. Users were motivated to contribute more and higher-quality data by a simple user interface to upload images, which then rewarded them with rapid results of information about “their” individual whales. Accessibility creates a public good as a resource for research, education, resource management, and science communication. In the existing NPPID dataset, 66% of all North Pacific humpback whale encounter data are publicly visible. Researchers and community scientists can explore migratory connections across the North Pacific via the web platform (Fig. 2). For research collaborators, this has inspired studies that would not have been possible without the large collective investment in building a platform and populating it with a comprehensive and contemporary dataset^50,51^. As of December 2022, the NPPID had contributed data to seven other collaborative peer-reviewed publications^13,37,38,52–55^. Accessible information about North Pacific humpback whale individuals has also proven very useful for resource managers, for example in tracking fishing gear entanglement cases, and individual identification and past sighting histories of dead or stranded whales^56^.

We recognize that including many actors and an open-science stance can add complexity to a collaboration^57^ with concerns for misuse of shared or public data^58^. Successful aspects of this collaboration bring opportunities but also pose two challenges that the collaboration must address: (1) How do we encourage contributing researchers to allow public visibility of data to allow the widest possible benefit, while ensuring data are used correctly in context, with proper credit preserved? (2) How do we simplify and clarify co-authorship policies to be effective, meaningful, and not so complex as to hinder publication?

An ideal collaboration builds datasets that directly answer present biological and management questions, and simultaneously creates data-sharing readiness. Data readiness for study of ecological change depends on both standardized repositories and aligned research interests^13,59,60^. The NPPID dataset has been successfully applied in this context, contributing to challenging management issues such as the US West Coast Dungeness crab fishery. Here, researchers can readily determine the proportion of whales in the Endangered Central American DPS^51,61–63^. The NPPID collaboration began with a MOA, offering co-authorship to contributors in a series of publications investigating humpback whale migratory patterns and population status in the North Pacific. Collaborators wishing to address additional research questions must seek permission from all relevant data contributors. While the communication required is a cost imposed on prospective studies, community is built around mutually beneficial collaboration. The MOA created an effective working group and context for this study through the completion of the specified series of publications. Future success will require clear use, sharing, and management policy, with oversight and funding maintained into the future.

### Data quality improved by accessibility

Accessibility adds value as part of the FAIR Principles for scientific data^45^ that guided this study design. Accessibility also serves the immediate practical value of improving data quality, consistency, and repeatability. Active collaboration and public access to data make knowledge gaps more visible and encourages effort to fill them^64^. With many eyes reviewing the dataset, from curious public enthusiasts exploring encounters of “their” whales or an area of their personal interest to research collaborators pursuing diverse lines of inquiry, an ongoing collaborative quality control process frequently detects data discrepancies. Happywhale user comments—over 5,700 as of August 2022—alerted NPPID data managers to enough errors that public accessibility to review might be considered as a systematic method of quality control, worthy of attention for its own value and efficiency.

All datasets will contain errors; more accurate image recognition, repeatedly applied, and review of data by diverse users will continually detect some, but not all errors. The SPLASH study estimated a 9–10% rate of missed matches using trained human matchers, the largest model error correction factor in the associated mark-recapture population estimate^21^. This kind of accuracy assessment rarely appears in photo-ID based mark-recapture studies, yet missed matches were detected in every dataset larger than 100 individuals involved in this study. Our finding of 331 false negatives in 7971 (4%) total individuals in the SPLASH study, when added to algorithm error rates for good-to-high quality images of 1–3%^42^, suggests the 9–10% error estimation was high by 3–4%. In our most accurately matched large dataset, the 2004–2020 whales of Glacier Bay National Park and Preserve, Alaska, missed matches accounted for only 0.15% (1 of 633 individuals, a first-summer calf to adult match with substantial fluke pigment change). All other datasets of more than 100 individuals showed from 2 to 11% detectable false negative missed match rates. Considering this range and other sources of error and bias, it is important to understand and account for limitations in any dataset, including ours.

### Effort bias and appropriate use

Ideally, a dataset should be created with its specific use in mind a priori, following a good data management plan^49^ with an optimized data workflow^65^. However, because we built a dataset gathered from post-SPLASH photo-ID archives and opportunistic efforts, standardization had to stand in for a priori data management plans. The effort was geographically and temporally heterogeneous, and any study design or interpretation of data must account for this to ensure appropriate use. It would be easy, for example, to falsely interpret the lower effort in the western North Pacific as evidence of smaller whale populations. Datasets cannot be assumed to provide an error-free documentation of humpback whale presence in the study area (i.e., devoid of effort bias); no clear rule can be set a priori to identify the appropriate application of an evolving dataset of this nature. It is therefore imperative that any potential data user actively engage directly with collaborating researchers to understand data limitations and potential. Data contributors can also be the primary data users, a group that will benefit from increased knowledge of and aptitude with the data management system built through Happywhale.

Because there could not be a comprehensive data collection plan across this large scale of a study area and time period, the full dataset might be considered opportunistic, a sum of collected efforts of dedicated research, research from platforms of opportunity, and community science contributions. Figure 4 demonstrates a large increase in data collection over time, elevated during the 2004–2006 SPLASH study, then building to similar levels from 2017 forward. Data collection rates have benefitted from many factors. These include: improvement in digital cameras, the growing popularity of whale watching, the 2015 establishment of the Happywhale platform, increased effort by many NPPID collaborators to capture fluke photos within existing field efforts, and the 2020 establishment and NOAA Fisheries funding of the SPLASH-2 program. The latter helped fund data collection efforts in poorly sampled areas, and infrastructure to support submissions to Happywhale. Our peak sample year was 2019, with 6,384 (21%) of 30,100 known North Pacific humpback whales documented. The COVID-19 pandemic interrupted both field research efforts and tourism in 2020 and 2021 (Fig. 4), though we believe sampling will recover and continue to increase.

### Building a successful collaboration

The NPPID study benefitted from the largely successful precedent of the SPLASH study both in providing a foundation of data (Fig. 4) and as a collaborative framework. The current study began at a time when new methods were needed to efficiently manage large volumes of post-SPLASH data, where separate research efforts were constrained by time-intensive visual matching of photo-ID datasets. Although the SPLASH study produced notable insight and remains frequently cited, and the catalog was made available online, the study was not intended to continue beyond 2006, and the online dataset was not built to facilitate photo-ID matching. The role of the NPPID collaboration agreement was to establish clear expectations and create an environment of openness, trust, transparency, and consistency. This context was necessary for research collaborators to feel comfortable sharing images and data that were products of many thousands of person-hours and costs in the field. Positive and useful feedback delivered by rapid results from image recognition efforts was also necessary. Researchers were enticed to join the collection in part by the instant gratification when most of their flukes immediately matched to known individuals; this was a welcome change from years of toil over visually matching isolated photo-ID datasets. Success was crafted by a combination of a high-quality product supported by solid guiding principles of Transparency, Responsibility, User focus, Sustainability and Technology (TRUST), to promote digital repository trustworthiness^66^. The idea behind these principles is that as a data repository, we must earn the trust of the community we serve and demonstrate that we are reliable and capable of appropriately managing the data we curate. Empowerment comes through this intentional framework, with a feeling of collective ownership rather than isolated possession. This then supports sustainable collaboration by creating active participation of research users.

As an ongoing, living dataset, the NPPID developed active, increasingly decentralized participation in ongoing data management with an intent to serve diverse needs in the research community. System development remains ongoing, with a focus on providing research collaborators with tools to become more directly involved with data management. This development reduces centralized data management costs, serves the real-time needs of collaborators, and benefits the dataset with local expertise, potentially detecting data issues that would not be recognized by remote data managers.

## Conclusion: sustainability and maximizing future value

The NPPID effort has established a single unified repository. This has been accomplished by reconciling all available research catalogs and ID nomenclature, and by aggregating all individual identities and encounter data into a state of data readiness unprecedented on a long-term and ocean-basin-scale. The first benefits are cost savings and organizational effectiveness. Particularly in well-sampled areas, data processing is revolutionized by immediate access to a fast and reliable photo-ID system. Collaborators reported that this “saves countless hours of manual visual matching, allowing us to get our data out in products, papers, and outreach more quickly” (JN) and “reduces lab time by 90%” (AS). However, collaborators face the challenge of how to maximize the present and future value of the NPPID dataset. A primary outstanding need is to create clarity for how researchers efficiently access, establish permission, and create sub-collaborations to develop further studies beyond the term of the NPPID collaboration.

System functionality was developed in a constant feedback cycle to accommodate progressively larger datasets through the study. This dataset appears to document most living humpback whales across the North Pacific Ocean basin, creating an abundance of data and inspiring an ambition to monitor populations in near-real time. With heterogeneous sampling effort over the study area, critical data gaps can be identified for understanding abundance and population structure. In addition, minimum sample sizes for reliable, robust population models can be established. Given the low cost of data storage, and if the incremental cost of each additional data point is driven to near zero, there is very little cost to overshooting a threshold of “enough” data.

Having now acquired sufficient baseline data for North Pacific populations in the face of a changing ocean, we aim for data readiness to understand the implications of ecosystem events on a timescale that benefits resource management. This study concentrates on humpback whales of the North Pacific, but the concept and methods can be extended to many species. Baleen whales are recognized to influence marine ecosystems on a massive scale^67^. In recognition of the concept of essential biological variables^68,69^, there is a need for marine observation and data at an ocean-basin-wide scale^70–72^. This dataset, the collaboration agreement, and the system established to create and maintain it can contribute to our understanding of essential ocean variables.

This study established an extremely cost effective and utilitarian information architecture, delivering an essential service for ongoing studies. If investment in collaborator engagement, upkeep, development, and data management continue, the future of this collaborative system promises great contributions to the understanding of North Pacific humpback whale populations. Sustainability will require a transition from the centralized efforts of a multi-year study to an established project at a stable institution with community ownership, oversight, and funding. We see this effort not as collecting and possessing a dataset, but as curating a public good for the betterment of science, education, and marine conservation. The FAIR and TRUST principles are central to guiding development, recognizing that accessibility requires more than just a data search feature via a web browser. To truly achieve full potential will require decentralizing data management to research collaborators, a shift that requires further system development, funding, user training, and commitment. Involving scientists in data management has evolved through time from a widespread disconnect^73^ to a current trend of ecological “big data” where data management is a necessary skill for ecologists, as has already happened with statistics and GIS^74^. We believe that establishing this scale-shifting dataset, given continued investment, will continue to improve understanding, awareness, stewardship, and respect for the North Pacific marine ecosystem.

## Supplementary Information

Supplementary Information 1.

Supplementary Information 2.

## Data Availability

The publicly viewable 66% of the full dataset used in this study, with ongoing additions and updates is available for exploration at www.Happywhale.com. All data are available with collaborator agreement to explore at Happywhale and in spreadsheet format. Please contact the corresponding author for discussion and permission. Approximately one-third of the dataset is public domain, but the collaborators believe that providing this partial dataset for open access download would be a disservice to the integrity of the full dataset.
