# Supplementary material for: A collaborative and near-comprehensive North Pacific humpback whale photo-ID dataset
Source: Sci Rep. 2023 Jun 23;13:10237. doi: 10.1038/s41598-023-36928-1 (PMC10290149; doi:10.1038/s41598-023-36928-1)
Supplement: Supplementary file 1 — Supplementary Information 1. [file 41598_2023_36928_MOESM1_ESM.pdf]

Simplified Memorandum of Agreement
North Pacific Humpback Whale Photo-ID Collaboration (NPPID)

GOAL

The development of **A Trans-Pacific Humpback Whale Matching Project** has two goals. The first goal is to determine the degree to which humpback whales move among different areas of the North Pacific. The second goal is to determine year-to-year population status and non-calf survivorship. By meeting these goals, the proposed study will refine knowledge of population structure and migratory movement using collections of humpback whale individual identification photographs held by individual researchers and organizations from selected regions of the North Pacific. The aim is to gather as large and deep of a North Pacific dataset as possible, therefore Happywhale requests all available digital images of non-calf humpback whale flukes from each research group.

BACKGROUND

This project is aimed at furthering the ongoing Comprehensive Assessment of North Pacific Humpback Whales being conducted by the International Whaling Commission, which endorsed these goals at the 2018 meeting of its Scientific Committee. Considerable work to address this question has been conducted by the SPLASH project (a North Pacific basin wide study called Structure of Populations Levels of Abundance and Status of Humpbacks), which collected and compared thousands of images. However, the SPLASH data and subsequent analyses included representation from some areas with small sample sizes. Data collected from these areas has significantly increased since SPLASH was completed in 2005 for the feeding areas and in 2006 for the breeding areas. Therefore, this project has a two-fold mission to include new data from underrepresented areas and add longitudinal data with the overall goal of improving the population assessment of North Pacific humpback whales.

AREAS OF COLLABORATION

Happywhale will compare images contained in catalogues provided by Happywhale and the members of the North Pacific Group using automated image recognition, with images managed within the Happywhale system. This provides for rapid automated comparisons of thousands of photos, thus greatly reducing the time required from labor-intensive manual matching. Image recognition technology is developing rapidly, and it is anticipated that improvements in

algorithm power will occur during the course of the study.

This collaboration will result in two published papers as outcomes from goals 1 and 2. Happywhale will use these papers as dissertation chapters as partial fulfillment of a PhD at Southern Cross University in Lismore, New South Wales, Australia.

ROLES AND RESPONSIBILITIES OF PARTNERS

Happywhale:

1. Any images provided by Contributors and the related matching results will be used exclusively by Ted Cheeseman/Happywhale studies of movement patterns, population status and non-calf survivorship described above. Ted Cheeseman/Happywhale will be lead author on the two manuscripts with publication (or in press) prior to the termination of this MOA.

2. Authorship will be made available to the members of North Pacific Group for any publication as an outcome of these efforts. Authorship will be based on meeting one of the following criteria: data acquisition, involvement in study concept/design, and/or data analysis/interpretation. Authorship format will be discussed among Partners and will depend on the journal policies. Authorship may be listed in a traditional format where a data contributor is offered co-authorship. Alternatively, contributors could be listed on a separate page inserted between the title page and abstract that includes their affiliations and funders/sponsors. Either way the goal is for the members of the North Pacific Group who have contributed to the manuscript to be recognized for their contributions upfront and not simply in the acknowledgements.

3. If any additional objectives or studies are identified for use of data contributed by the North Pacific Group beyond Ted Cheeseman/Happywhale studies of movement patterns, population status, and non-calf survivorship, they will not be pursued without written consent of the principal contact from each North Pacific Group member/research organization whose data would be included. This includes any product that synthesizes any data provided by the Contributors (including journal manuscripts, technical reports, International Whaling Commission (IWC) reports, photo catalogues, media products, etc.) without the Partners discussing and agreeing to the details of content and authorship.

4. In regards to any report to the IWC arising from this project, all Contributors will be provided the opportunity to read and comment on the draft a month or more before its presentation. If any Contributor does not agree with the emphasis or interpretation of the collective data they are entitled to author a paragraph stating their view, which will be included in the report.

5. The use of any data from North Pacific Group members that was included in the pre-2004 archive of photographs (non-digital) of humpback whales transferred from the National Marine Mammal Laboratory to Happywhale also falls under this MOA agreement as a default. However, contributors may include additional restrictions to the use of the pre-2004 data.

6. Feedback on matching results involving data from Contributors will be provided rapidly via an automated system within Happywhale, which will also give Contributors details of the sighting histories of the whales concerned (subject to any restrictions placed by the data contributors).

7. Happywhale will communicate with Contributors at six-month intervals, providing a status update on the project.

8. Happywhale will not share images from Contributors with third parties or the public without prior written consent from the Contributor.

9. Any matching result(s) from Happywhale's analysis using data from Contributors that may warrant a separate publication (for example, a particularly interesting, first or rare, match between two areas) will first be communicated to the specific data contributors before results are disseminated to other members of North Pacific Group. The relevant contributors will be given the right of first refusal to publish this information as primary authors. Furthermore, how the results are provided to media or made public needs to be agreed by the Partners involved.

10. Data provided by Contributors will be stored within the Happywhale system, however within this system these records will be assigned to a 'North Pacific Humpback Whale Photo ID Study' media permissions group that may have restricted access, based on the determination of individual Contributors, with only the Partners allowed access to these data. Data contributed by North Pacific Group members will only be visible to the public or to any other individuals or institutions with the explicit permission from the individual Contributor. Happywhale account permissions for Contributors will be established for designated members which will allow them to view and have access to the group's restricted data alongside any public data on the Happywhale site.

North Pacific Group:

Contributors will submit photographs and associated metadata to Happywhale in a timely manner.

1. Contributors will respond within one month to communications from Happywhale regarding feedback on results, manuscripts, and other topics related to the project goals.

2. Contributors may share their own data with third parties and the general public without limitation and at their own discretion throughout the project.

3. Lead authorship will be available to a member of the North Pacific Group who initiates a study/analysis as an outcome of the data comparison outside of Happywhale's analyses who takes responsibility for preparing and following through with a manuscript for publication. If a Contributor decides to pursue this path a brief proposal and timeline will be submitted to Happywhale and North Pacific Group members for review. Although all Partners will review the proposal, the decision to agree to the use of the specific data involved in the analysis will be made by the relevant data contributors.

4. If a Contributor has a prior agreement with Happywhale the initial agreement will take precedence over this MOA as agreed upon by Happywhale and the Contributor.

USE OF INTELLECTUAL PROPERTY

The parties agree that any intellectual property, which is jointly developed through activities covered under this MOA and all other intellectual property used in the implementation of the MOA will remain the property of the party that provided it. This property can be used by either party for purposes covered by the MOA but consent will be obtained from the owner of the property before using it for purposes not covered by the MOA.

TERMINATION

Any Partner (including individual Contributors) may terminate this MOA and any related agreement at any time and for any reason by giving 30 days prior written notice to the other Partner; provided, however, that in the event either Partner fails to perform any of its obligations under this MOA, the other shall have the right to terminate this MOA and any related agreement immediately upon written notice. If a Contributor decides to terminate their relationship with Happywhale, the initial data will still be available to Ted Cheeseman for use in his dissertation as long as Happywhale has met the obligations under this MOA.
