# Supplementary material for: A collaborative and near-comprehensive North Pacific humpback whale photo-ID dataset
Source: Sci Rep. 2023 Jun 23;13:10237. doi: 10.1038/s41598-023-36928-1 (PMC10290149; doi:10.1038/s41598-023-36928-1)
Supplement: Supplementary file 2 — Supplementary Information 2. [file 41598_2023_36928_MOESM2_ESM.pdf]

 DOI : 10.1038/s41598-023-36928-1

Acknowledgements for A collaborative and near-comprehensive North Pacific humpback whale photo-ID dataset

Happywhale would like to acknowledge each of the thousands of image contributors who create the strong community of marine mammal enthusiasts who make Happywhale a resource for ocean conservation science. The authors wholeheartedly thank the whale watch guides, leaders, photographers and tour company owners who make it part of their mission to share and educate the world about whales and ocean life. A full list of contributors to the North Pacific dataset in Happywhale is at the end of this document. Happywhale wishes to acknowledge funding support from Cheesemans' Ecology Safaris, NOAA Fisheries, Pacific States Marine Fisheries Commission, Cascadia Research Collective, University of Alaska Southeast, The Marine Mammal Center, the Hurtigruten Foundation, Booking Cares, Viking Expeditions, Lindblad Expeditions, Amber Group, Defenders of Wildlife, and all individual supporters of Happywhale.

Daniel Palacios and Craig Hayslip (Oregon State University, Marine Mammal Institute, Whale Habitat, Ecology, and Telemetry Laboratory) acknowledge the financial support from the U.S. Navy's Office of Naval Research; the Tagging of Pacific Pelagics program; the Steve Irwin Foundation; the Pacific Life Foundation; the Makana Aloha Foundation; the Navy's Pacific Fleet Commander (via CESU Cooperative Agreement No. N62473-19-2-0002); and private donors to the Oregon State University Marine Mammal Institute Endowment. Photographs were collected under NMFS Marine Mammal Protection Act/Endangered Species Act Research Permit Nos. 841 (1995 to 1998), 369-1440 (1998 to 2004), 369-1757 (2005 to 2008), 14856 (2014 to 2018), and 21585 (2019) issued to Bruce Mate.

Okinawa Churashima Foundation thanks S. Ozawa, Y. Miyamura, H. Miyahira, K. Tamura, and K. Miyahira for the data collections in Okinawa.

Cascadia Research Collective would like to thank the many contributors and participants in the SPLASH study that was an important foundation and dataset to the current effort. We thank NOAA for funding some of the data collection especially off the US West Coast, Central America, and southern Mexico in recent years as part of their support for SPLASH-2.

All data in the Mariana Islands were collected under NMFS MMPA permits 15240, 20311, 21482 and the CNMI DFW permits 03086-15, 03292-16, 03564-17, 03752-18. Funding was provided by the U.S. Navy (Commander, U.S. Pacific Fleet) and the Pacific Islands Fisheries Science Center. We thank Allan D. Ligon and Adam C. Ü for their significant contribution to PIFSC fieldwork in the Marianas and NAVFAC/HDR for their contribution of images from their survey of FDM in 2020.

UNAM thanks M.J. Vázquez-Cuevas and the many students from UNAM that for years have worked curating photographs and at sea.

Pacific Whale Foundation acknowledges that funding was provided by the members of Pacific Whale Foundation and private donors. Photographs were collected under MMPA/NMFS permits 323, 399, 565, 812, 982, 468-1574, 13427, 16479 and 21321. We acknowledge the support of many team members, both staff and volunteers, that contributed to PWF's long-term humpback whale research and in particular thank Abigail Machernis, Florence Sullivan, and Elizabeth Beato for their assistance curating the PWF catalog and uploading it to Happywhale.

University of Alaska Southeast (UAS) lab of Heidi Pearson acknowledges photographs were collected under NMFS permits 14122, 18529, and 20688 and UAF IACUC protocols 464648 and 1604256. We thank Chris Pearson and the many UAS undergraduates who helped with data collection and analysis. Funding was provided by Alaska EPSCoR, the University of Alaska Foundation Angus Gavin Migratory Bird Research Grant, Alaska Sea Grant, BLaST, and NMFS through a grant to the Pacific States Marine Fisheries Commission.

The North Coast Cetacean Society thanks the Save Our Seas Foundation, Willowgrove Foundation, the Department of Fisheries and Oceans, Donnor Canadian Foundation, all private donors, volunteers and staff and the Gitga'at and Kitasoo/Xai'xias First Nations. Photographs were collected under research permit MML-43.

Glacier Bay National Park & Preserve and UAS Sitka acknowledge all data were collected under NMFS ESA/MMPA permits 600, 845, 945-1499, 945-1776, 15844, 21059, P263, P317, 473-1700, 473-1700, 14122, and 18529. We acknowledge the many Park staff and volunteers who helped collect and process the data over the decades of study.

Ester Quintana-Rizzo thanks the funding support of various institutions including Cascadia Research Collective; Fondo Nacional de Ciencia Tecnología, awarded by the Consejo Nacional de Ciencia y Tecnología, through the Secretaria Nacional de Ciencia y Tecnología (Fodecyt 85-2007 Project), Cetacean Society International, Sarasota Dolphin Research Institute, Idea Wild, Defensores de la Naturaleza Foundation, and SPLASH 2 coordination provided by NOAA Fisheries West Coast Region and NOAA Fisheries Office of Protected Resources

Whales of Guerrero thanks the communities of Barra de Potosí and Zihuatanejo in Mexico for their assistance and support, the dedicated scientists, educators, community members and expedition guests who collected the data with us (in particular, Arturo Mellín, Terra Hanks, Victoria Pouey Santalou, Claudia Auladell Quintana and Cristina Martin, Idea Wild, Cetacean Society International, USFWS/Semarnat Wildlife Without Borders, National Geographic Society, Lighthouse Foundation, Norcross Wildlife, Oceanic Society, SEE Turtles, NOAA, Cascadia Research Collective, Smultea Environmental Sciences, Mysticetus, Adobe, Luis Medrano Gonzalez at UNAM, and the private donors who made this work possible. Data was collected under research permits: SGPA/DGVS/12143/16, SGPA/DGVS/011899/17, SGPA/DGVS/010770/18

Ecología y Conservación de Ballenas, A.C. thanks FIBB Catalog contributors: Ecotours Vallarta, Vallarta Natours, Instituto Tecnológico de Bahía de Banderas, CRIP-INAPESCA, Vallarta Adventures, Orca de Sayulita, Oceanfriendly and Cielo Abierto who collected data. We also want to thank Fundación Ecológica BIOMAR, Stanley W. Ekstrom Foundation, Junghanns, Opequimar Centro Marino, PV Marine, Ecotours Vallarta, October Hill Foundation and many other private donors for their kind contributions to make this project possible.

NOAA Hawaiian Islands Humpback Whale National Marine Sanctuary would like to acknowledge the support of staff and volunteers that contributed to the Sanctuary's long-term humpback whale fluke catalog; and the funding support provided by Whale Trust (via Whale Tales awards), the Volgenau Foundation, Deborah and Michael Rybak, Whaleman Foundation, and many private donors that make the catalog possible. Special thanks to Rachel Finn for curating the Sanctuary's catalog.

Photographs were collected under MMPA/ NMFS permits 14682, 15240, 20311, 932-1489, 932-1905 and 18786.

Alaska Whale Foundation images were obtained under NOAA/NMFS Research Permits No. 0716-1456, 716-1705, 14599 and 19703.

Whale Trust acknowledges the many supporters who have funded our research over the years, ultimately allowing our photo-id dataset to be shared. We thank Cathy Maxwell and Haley Robb for their efforts in helping to manage our photo-id catalog with Happywhale submissions. These images were collected under NMFS-NOAA permits: 753, 987, 13846, 19925.

For the Philippines, we thank WWF-Philippines, all the volunteers and researchers of BALYENA.ORG particularly Elson Q. Aca, Shotaro Nakagun, Timothy Gardner and Takeya Sakamoto. We are grateful for the funding support from Ocean Park Conservation Foundation – Hong Kong, The Rufford Foundation, Whale Trust and all our donors.

Marine Education and Research Society (MERS) acknowledges this work would not be possible without the contributions of the ecotourism, research, and conservation communities of northeastern Vancouver Island, and the staff, volunteers, and supporters of the Marine Education and Research Society (MERS). Funding was provided by Fisheries and Oceans Canada and the North Island Marine Mammal Stewardship Association. Data collected under research licenses MML-42 and MML-57.

Funding for the Fisheries and Oceans Canada (DFO) humpback catalog and database was provided by the DFO Species at Risk Program and the Parks Canada Agency. DFO would like to thank the many individuals and organizations that have contributed to this effort over the years.

Marine Megafauna and Fisheries Research Group (MMAPE) thank Ricky Rebolledo and Vicotira Jansen for all their generosity in lending us a zodiac to be able to take pictures in the Revillagigedo archipelago. We want to say thank you so much to Sea Shepherd Conservation Society, and all who participated as part of the crew of R/V Martin Sheen and R/V White Holly, during the research campaigns to the Revillagigedo archipelago from 2019 to 2021. And especially we want to thank Eva Hidalgo, Andrea Bonilla, John Payne, Derek Kimball and JP. Also, we thank all the students who supported both field and photo editing work. We also thank the staff of the Revillagigedo National Park, particularly Erendida Frías for all her support in organizing the visits to the park. We thank the SEP-CONACYT #204778 project that financed part of the expeditions to Revillagigedo from 2019 to 2021. Finally, to the academic department of fisheries engineering of the UABCS, to the Dirección General de Ecología del Municipio de Los Cabos and to the staff of Cabo Expeditions, particularly Diego Ruíz and Oscar Ortiz, who supported us during our photo-ID work in Los Cabos.

Joëlle De Weerdt (Association ELI-S) thanks the private donors and the institutions who provided funding for this research including Cascadia Research Collective, Idea Wild, Cetacean Society International, Garmin, Joanna Toole Foundation, and Rufford Foundation. Special thanks to the captains and all the fieldwork assistants who helped collecting the data with us. The research was done under research permits of MARENA: DGPNB-IC-001-2016/ 009-2018/ 006-2019 / 011-2020 / 011-2021.

Hawaii Marine Mammal Consortium acknowledges photography and fieldwork by Suzanne Yin, Susan Rickards, Adam Frankel, Kim New and others whose images were obtained under NOAA/NMFS Research Permits 774-1437, 782-1719, 1127-1921, 15330, and 20605.

Funding from the University of Hawaiʻi at Manoa and the Office of Naval Research Department of Defense (Grant # N00014-19-1-2612) made this research contribution possible from the Marine Mammal Research Program at the University of Hawaii at Manoa.

Happywhale would like to acknowledge the following photo and data contributors for the North Pacific dataset:

Photo-contributing scientists and research groups:
Bernardo Alps, Diane Alps, Carlos Alvarez, Dave Anderson, Eric Angel Ramos, Lisa Angle, Aquarium of the Pacific, Eileen Avery, Annie Douglas Baird, Robin Baird, Ken Balcomb, Daniel Bianchetta, Nancy Black, Jim Borrowman, Elliott Breslar, Vladimir Burkanov, Ethan Bushey, Dave Cade, Dominique Camacho, Salvatore Cerchio, Todd Chandler, Channel Islands NMS (CINMS) Naturalist Corps, Casey Clark, Marilyn Dahlheim, Luciano Dalla Rosa, Rene Devito, Bethany Diehl, Discovery Whale Watch research, Don Dolittle, Carlos Andres Elgueta Godoy, Dave Ellifrit, Candice Emmons, Ma. Eugenia Rodríguez Vázquez, Joe Evenson, Joseph Evenson, James Fahlbusch, Erin Falcone, Holly Fearnbach, Debbie and Mark Ferrari, Michael Fishbach, Ian Flynn-Thomas, Pieter Folkens, Karin Forney, Hillary Foster, Adam Frankel, Frank Garita Alpízar, Kathi George, Don Gillies, Brian Gisborne, Paulina Godoy y Carlos Aguilera, Bonnie Gretz, Geoff Grow, Grupo de Investigación de Mamíferos Marinos, Chris Hamilton, Jeff Harris, Elizabeth Henderson, Shoal Hollingsworth, Kathy Hough, Jessie Huggins, Yulia Ivashchenko, Laura J. Morse, Shirel Kahane-Rapport, Josh Kaye-Carr, Eric Keen, Sara Kerosky, Thomas R. Kieckhefer, Amber Klimek, John Kuizenga, Barbara LaCorte, Daniel Laggner, Marc Lammers, Lei Lani Stelle, Ingermarie Laursen, Scott Leon, Ron LeValley, Steve Lewis and Rachel Myron, Dave Lott, Emma Luck, Amber Luvmour, Marine Life Studies, Eric Martin, Beth Mathews, Cathy Maxwell, Carolyn McCleskey, Gitte McDonald, Josh McInnes, Elizabeth Mitchell, MMOBiDiC, Nora Moloney, Jim Nahmens, Anne Nelson, Flip Nicklin, North Gulf Oceanic Society, Marie O'Shaughnessy , Dan Olsen, Lenin Oviedo, Kim Parsons, Doug Perrine, Robert Pitman, Programa Nacional de Conservación de Cetáceos (ESA), Jennifer Quan, George R Torralba, Chris Rado, Jessica Redfern, Alan Reitsch, Heather (Vukelic) Riley, Brenda Rone, Hiram Rosales Nanduca, Elske Rotshuizen, Phil Rouget, Alexis Rudd, Peter Rudolph, Bart Rulon, Marco Saborio, Dan Salden, Doug Sandilands, Kate Sardi, Oksana Savenko, Jim Scarff, Ernst Schneidereit, Greg Schorr, Alisa Schulman-Janiger (California Killer Whale Project), Dan Shapiro, Charlie Short, Glenn Shuart, Andrea Siatkowski, Michael H Smith, Clark Snodgrass, Lisa Spaven, Peggy Stap, Gretchen Steiger, Alison Stimpert, Wendy Szaniszlo, Isidore Szczepaniak, Angela Szesciorka, Jenn Tackaberry, Barbara Taylor, Kate Thomas, Leigh Torres, Marcel V. de Morais, Alexandra Vanderzee, Janice Waite, Eric Ward, Jane Watson, Bridget H. Watts, Sophie Webb, Daniel Webster, Bill Weinerth, Caroline Weir, Monika Wieland Shields, Ulrike Wolf, Manami Yamaguchi, Suzanne Yin, Eden Zang, Sachie Ozawa, Naoto Higashi, Senzo Uchida, Kiyoteru Toyama, Yukifumi Miyamura, Hisao Miyahira, Keiko Tamura, Kiyohide Miyahira, Okinawa Churaumi Aquarium staff

Photo-contributing community scientists:
Kevin Abernathy, Robin Abernethy, Caitlin Abraham, Julie Abrams, Celia Ackerman, Sarah Acosta, Colin R. Adair, Jenn Adair, Jessica Adams, Paul Adams, R.J. Adams, Sheree Adams, Jenn Addison, Ellen Adelman, Sanjay Adkar, Andrew Adler, Marilyn Adler, Christian Adlhart, Oceanside Adventures, Vallarta Adventures, Sion Agami, Robin Agarwal, Helen Ahern, Artie Ahier, Jay Ailworth, Bruce Aitken, Kak Alak, Thomas Alcorn, David Alders, Michelle Aldrich, Caryn Aleo, Margaret Alerding, Heidi Alexander, Maria Alexis, David Alfaro, Nelson Enrique Alfaro Hernandez, Mohammad Ali Pasha, Salim Ali Siddiq, Andromeda Alighieri, Ben Allen, Darrin Allen, Karen Allen, Paul Allen, Cindy Alley, Liz Allyn, Watt Alston, Susan Altshuler, Jennifer Alvarez, JoLynn Alvarez, Michael Alves, Nadia Aly, Gerry Ambury, Heros Amerkhanian, Cindy Amistoso, Peter Amster, John Amussen, Avi Anand, Blain Anderson, Jacob Anderson, Kiersten Anderson, Ron Anderson, Tom Anderson, Susan Andersson, Kelvin Andow, Tisa Annette, Lardeau Annick, Shelly Antonali-Tinsley, Greg Aranea, Thais Arata, Fernando Arcas, Joe Arceneaux, Diego Arciniega, Duncan Armour, JC Armstrong, Howard Arndt, Chris Arnold, Jen Arnold, Michael Arrighi, Rodney Artiles, Tammy Ash, Nick Ashby, Jeannette Ashlin, Laurel Ashlock, Carl Ashton, Anna Astafurova, Allen Atkinson, Jen Atkinson, Jessica Aubry, Sean Aucoin, Jim Audley, Kristi August, Brian Auman, Eric Austin Yee, Jan Austin, Julie Austin, Linda

Averett, Burak Ayday, Mark Edwin Ayre, Chuck Babbitt, Stephen Baca, Don Baccus, Lars Backstrom, Angela Bacon, Tammera Badano, Lee Bagby, Dana Bagshaw, Bob Bailey, Lynnette Bailey, Paul Bailey, Michael Bain, Mike Baird, Jose Baires, Raminder S Bajwa, Martha Baker, Erik Bakke, Sanne Bakkers, Kelly Bakshi, Bruce Baldi, Anthony Baldo, Kevin Baldwin, Lisa Baldwin, Sara Baldwin, Susan Balkema, Abbie Balland, Mike Bamshad, Paul Bandall, David Banks, Caitlyn Bannister, Kerry Barbour, Jack Barkowski, Jay Barlow, Matthew Barnes, Lory Barra, Salvador Barraza Delbarco, Joel Barrett, Lisa Barrett, Danica barron, Dave Barry, Lisa Barry, Wesley Barry, Peter Barto, Gina Barton, Joe Barwick, Jessica Bas, Robert Basaraba, Kirstie Basford, Heather Baskey, Tanya Bass, Tanya Baston, John Batchelder, John Bateman, Marcella Battista, Jeff Baublits, Alisa Baum, Sara Baumgartner, Kym Bausch, Diego Bautista, Carrie Baxter, Chelsea Baxter, Melissa Baxter, Siri Baxter, Ryan Bayliss, Cathy Beard, Doug Beasley, Laurie Beaubien, Rick Beaver, Vicki Beaver, Katie Bechtel, Ann Beck, Steph Beck, Cindy Becker, Hilary Becker, Ivo Becker, Joe Beckert, Brittany Becklund, Peter Bedrossian, James Beedle, Richard Beeler, David Beeninga, Renske beerkens, Christie Beetz, James Begeman, Rich Behn, Chelsea Behymer, Renee Beitzel, Cindy Belko, Danny Bell, Bret Bellevue, Mitchell Belser, Malory Benedetti, Chris Benesh, Chuck Bennett, Kathy L Bennett, Amanda Bennewitz, Laura Benson, Gena Bentall, Veronica Benton, Heather Benz, Scott Benz, Lori Beraha, Ferd Bergholz, Carey Bergman, Marcus Bergström, Andrew Bergstrom, Marcus Bergström, Bryan Berkowitz, Don Bermant, David Bernal, Carin Berolzheimer, Shamus Berry, Sierra Bertini, Allan Besson, Laura Beth, Chris Beukenkamp, Keshav Bhanot, Domenic Biagini, Alain Bidart, Chris Biertuempfel, Daniel Biggs, Denise Billen-Mejia, Rayne Billings, Eric Bindseil, Emma Bingham, Biocean Wildlife Encounters, Jordan Bird, Paul Bishop, Piper Bishop, Brendon Bissonnette, Brent Bitterman, Jeff Bjornson, Frank Blache, Jill Black, Mackenzie Black, Maddie Black, Ron Blackard, Nick Blackburn, Cynthia Blackman, Mary O. Blackwell, David Blaen, Anna Blake, Johanna Blakley, Teresa Blase, Jamie Blattmann, Olivier Blaud, Dawn Blauser, Kurt Bleacher, Bridget Bleshenski, Annalese Bletsch, Sophie Blews, Max Block, Fran Bloom, Jeff Blum, ciaran blumenfeld, Elliott Blumenthal, Barbara Hall Blumer, Vicki Boardman, Carl Boast, Dawn Boatright, Kelly Boatwright, Haley Bochicchio, Carly Boddy, Nicki Boggs, Sarah Bohn, Mael Bojórquez, Ben Boline, Annette Bombosch, Gabe Bonde, Bladimir Antonio Bonilla Cortez, Carlos R Bonilla Ruz, Jan Booch, Shari Bookstaff, Khemarat Boonyapaluk, Sabrina Boquet, Glenn Borchers, Luciana Fortes Borelli Costa, Courtney Boren, Nicole Borger, Vanuza borges, Sheri Boroda, Sylvia Borunda Firth, John K. Borys, Jr., Kerry Bostrom, Brian Bottomley, Lisa Bourgault, Jim Bourne, Tori Bowe, Ginger Bowman, David Boyarski, Dina Boyer, Shavawn Boyer, MaryAnn Boylan, Heather Boyle Dimitrievski, RT Bozarth, Dana Bozich, Garrett Bozzo, Larry Bradfish, Daniel Bradford, Chelsea Bradley, Thomas Bradley, Wes Bradley, Chris Brady, David Brakebill, John Brandon, Rachel Bratlie, William Brawley, Sam Bray, Douglas Bready, Rob Breakiron, Cathy Breining, Tom Bremer, Chris Brendemuhl, Lisa Brendle, Capt Frank Brennan, Elliot Breslar, Evelin Breuers, Julie Brewer, Van Brewer, Cheyenne Brewster, Chalon Bridges, Kerri Brimmer, Mike Brink, Thor Brisbin, Jane Bristow, Evan Brodsky, Stan Brody, Angie Broennimann, Anieke Brombacher, Keith Brooks, Mariela Brooks, Dustin Brookshire, Jack A Broom, Audra Brousseau, Aimee Brown, Dan Brown, David Brown, Donald Brown, Ena Brown, Eric S Brown, Fletcher Brown, Nic Brown Photo, Nick Brown, Tara Brown, Wayne Brown, Terry L. Brown and Jude Abrams, Bryan Brubaker, Emily Bruce, Brittany Bruffey, Julie Brusca, Peter Bruton, Ashley Bruursema, Roger Bryenton, Paul Buck, Keith Buckingham, Justine Buckmaster, Todd Buczyna, Julie Buderus, Michael Buehler, Anita Buehlmann, David Buettner, Per Nikolaj Bukh, Andrew Bunnell, Linda Burback, Nancy Burbott, Alexander Burdin, Kourtney Burger, Florian Bürger, Raina Burke, Dustin Burkhouse, Margaret Burks, June Burlingame Smith, Mary P.K. Burns, Taylor Burns, Taylor Burtch, Alyce Bush, Rick Bush, Scott Bush, Adolfo Bustamante Lizárraga, Alicia Buzzard, Linda Buzzini, Karen Byars, Jayleen Bydlon, Vern Byggdin, Laurie Byington, Jamie Byrne, Cameron Byrnes, Wendy Byrnes, Virginia Caballero Slim, Jessica Cabrera-Sarduy, Jenny Caldwell, Mary Caldwell, Kandy Callaghan, Marcie Callewaert, Luis Tadeo Camacho Ledesma, Robert Camp, Dawnya Campbell, Ian Campbell, Ken Campbell, Kristin Campbell, Pamela Campbell, Sherry Campbell, Terry Canavan, Natalie Cannatella, Holly Cannon, Mae Elise Cannon, Carol Cappadonna, Capt. Dave's Dolphin & Whale Watching Safari, Captain Zodiac, Courtney Carballo, Gustavo Cárdenas Hinojosa, Jacky Carleton, Alyssa Carlson, Betsy Carlson, Dennis and Tina Carlson, Adam Carlton, Jeff Carlucci, Robyn Carmel, Rick Carpenter, Eric Carr, Megan Carrow, Kristie Carruthers, Lilian Carswell, Amy M Carter, Danielle Carter, Donna Carter, Greg Carter, Ted Caryl, Brooke Casanova, Robyn Case, David Cashbaugh, Thomas Caskey, Melvin Castaneda, Michael Castillo, Robert Castle, Zoe Castle, Ivan Lopez Castro, Ben Caswell, Brian Catania, Kelly Cates, Dean Catton, Emily Cavedon, Daniel Cayton, David Cervera, Sandra Chafee, David Chamberlin, Erin Chandler, Rodney Chandler, Alan Chang, Leo Chang, Jonathan Chapman, Tess Chapman, Mark Chappell, Ron Charbonneau, Stephanie Chastain, Erica Chavez, Freddy Chavez, Joe Chavis, Maxim Cheeran, Doug Cheeseman, Gongyao Chen, Jiyu Chen, Lancy Cheng, M N Cheng, Jonathan Cherney, Michelle Cherry, Holly Cheshier, Garrett Chew, Francine Chick, Shannon Chicoine, Gabriella

Childers, Lucita Chin, John Chiovarou, Casey Cho, Brooke Chodzinski, Seemant Choudhary, CHP Golden Gate Division Air Operations, Denny Chretien, Jonathan Christ, Emily Christensen, MaryBeth Chruden, Chris Cilfone, Tim Claes, Gina Clapp, Lione Clare, Justin Clarey, Brian Clark, Cecilia Clark, Julian Clark, Marianne Clark, andrea clarke, Chris Clarke, Emily Clarke, Rolf Clarke, Brent Clayton, John Clear, William Clements, Kris Clifford, Amanda Cline, Jill Clogston, Kathryn Clouston, Tom Coates, Chris Cobb, Jennifer Cobb, Rhonda Cobb, John Coffey, Kayleigh Coffey, Julie. Coffin, Joshua Cohen, Sheri Cohen, Laurie Colbeck, Amanda Colbert, Debbie Colbourne, Ian Colle, Drew Collins, Peter Colombo, Danielle Colosimo, Erik Combs, Brian Congdon, Denielle Conley, Anne Conlon, Lucia Conner, Jake Connolly, David Conroy, Ellen Conway, Abriana Cook, Paul Cook, Sarah Cook, Kathy Cookman, Bill Cooney, Charlie Cooper, Shaun Cooper, Taylor Cope, Kristen Coppola, Kira Corcoran, Rob Cordes, Joanna Corey, Lila Corkish, Juliana Cornett, Mark Andrew Cornish, Kristi Cornwell, Joseph Coronado, Gwiin Correa, Grace Corrigan, Caren Costley, Jim Cotton, Michael Cottrell, Paul Cottrell, Michael Couffer, Kelly M Coursey Gray, Tony Coward, Crystal Cowles, Chad Cox, Dana Cox, Jared Cox, Mark Cox, James Coyer, Andrew Crabtree, Tracey Cragg, Emily Cramer, Valerie Cramer, Allan Crandell, Hannah Crazyhawk, Lucas Cressler, Aaron Cretin, E Darrell Crisp, Kim Crisp, James Croan, Stacey Crofoot, Douglas Croft, Christian Crook, Mariann crooks, Helen Cross, Lee Crosson, Keely Crowder, Jessica Crowe, Gabriella Crowley, Sanctuary Cruises, Haide Cruz Villagran, Cserhati Csanad, Jim Cubbage, Teresa Cue, Michael S Cugno Jr, Crystal Cullen, Lisa Culp, Jennifer Cummings, Kate Cummings, Shea Cummings, Amanda Cummins, Sandra Cunha-Rocha, Tim Cunnell, Jeanne Cunningham, Cherish Curtis, Lindsay Curtis, Susan Curtis, Ashley Curtiss, Ali Curtner, Ashlee Cushing, Chris Cutler, Andy Cvengros, Sarah Czaplewski, Peter D'Angelo, Rachel Daddona, Abigail Dahl, Sandra Dake, Jim Dalton, Dana Wharf Whale Watching, Tatyana (Taz) Danahy-Moore, Caitlyn Dance, Arabella Dane, Juan Daniel Negrete Uribe, Conrad Dark, Nikhil Dass, Alison Davenport, Calley Davenport, Pat Davi, Shaun Davidson, Stu Davidson, Alex Davies, Mike Davies, Scott Davies, Susanne Davies, Carrie Davis, Chasen Davis, Chris Davis, Crystal Davis, Cyndy Davis, Doug Davis, Elizabeth Davis, Jessica Davis, Joe Davis, Kimberly Davis, Larry Davis, Maddi Davis, Roger Davis, Andrew Davison, Tiffany Davister, Andy Day, Michael Day, Rod Day, Karla De La Peña Valdes, Milton De Sousa, Eric De Torres, Mark Deakos, Sami Dean, Denis Dean Roth, Kim DeBaere, Jonathan Decker, Kristen DeCrausaz, Patrick Dee, Volker Deecke, Diane DeHaas, Nico Dehaerne, Chase Dekker, Alicia Del Toro, Kalasara Del Viscovo, Josh DeLeenheer, Jean-Louis Delezenne, Tracey DellaVecchia, Eli Delnore, Darrel DeLong, Michelle Demery, Dan DenDanto, DeAunne Denmark, Erin Denmark, Fiona Denney, Laryssa Densmore, Kyle Denson, Grace Denton, William Dereski, K.C. Dermody, Mike Desiderio, Tracy Desiderio, Jody Desin, Kandice Devenney, Todd Devlin, Marsha Devot, Kaelyn DeYoung, Mireya Diaz, Gabriela Diaz Erales, Claudia Diaz Guzman, Thais Dib Frias, Diane dickey, Kristie Dickinson, Ben Dickmann, Gabi Diedrich, Karen Dienhart, Tyania Diffin, Marsha Dillard, Amber DiLuzio, Jack DiMarchi, Duy Dinh, Linda Dinkelman, Roger Diseker, Paul Dixon, Will Dixon, John dockerty, Steve Dodd, Diana Dodson, Christine Doerr, Holly Doerr, Sybil Dogotch, Mike Doherty, Sean Doherty, Mark Doing, Richard W Dolan, Toni Domingo, Regina Domingo Esgleas, Aislinn Domínguez Ibarra, Johanna Domise, Mike Donald, Susie Donaldson, Danielle Dong, Ryan Donnelly, Mason Donny, Ann Donovan, Chris Donton, Trina Dopp, Srih Dore, Tracy Dorman, David Dovalina, Joseph Dowdy, Lynn Dowling, Natalie Downe, Deirdre Doyle, Brianna Doyon, Joshua Dragstedt, Jayme Drainville, Wendy Draper, Tammy Drazkowski, Susan Drelich, Jennifer Dreyer, Lauren Drown, Jessi Drum, Maurice Druzin, Grant Du, Chip Duden, Kecia Duffy, Patrick Duggan, Katie Dunbar, Melissa Duncan, Aimee Dunlap, Amanda Dunman, Annamarie Dunn, Craig Dunn, Rick Dunning, Tim Durham, Chris Dutton, Martin Dvorak, Nicole Dwyer, Andy Dyson, Ken Dzinbal, Katherine Eago, Darren Earl, East Meets West Excursions, Shannon Easterly, Greg Easton, Jessica Eberle, Phil Echelman, Tyler Eddy, Susana Ede Emilia De los Santos Reyes, Ciera Edison, Diane Edmonds, James P Edmonds, Walter Edmondson, David Efros, Bill Eggbeer, kelsey eggert, Brad Eichhorst, Bill Eikeland, Ben Eisenstein, Walter Ejnes, Karyn Ekola, Neal Elbert, Kathy Elder, Elwin Elias, Lynda Elkin, Ryan Elledge, Vickie Elledge, Clara Elliott, Jody Elliott, Graeme Ellis, Cindy Elliser, Katie Ellyn Smith, Stephen Elser, Olaf Elton, Lyn Emerich, Mallory Emerson, James Emert, Craig Emery, Craig Emmerich, Brian Emmett, Energía del Pacífico, Chris Eng, Libby English, Nick English, John Epperson, Cheyenne Epps, Kristine Erickson, Molly Erin Barrett, Katherine Ernst, Adam Ernster, Greg Eskew, Sarah Eslinger, Sherry Essmann, Andre Estevez, Albert Esther, Luis Estuardo Rios, Gautam Ethiraj, David Etzold, Caroline Evans, Chelsea Evans, Greg Evans, James Evans, Robyn Evans, Zena Eve Samuelson, Richard Evers, Jackie Everwine, Whale Facimar, Andre Fahrni, Jeff Fairbanks, Susan Fajardo, Brian Falconer, Ruth Falla, Cross Family, Maureen Fan, Kim Fanning, Marie Faraut, Dan Farbman, Tiffany Fare, Tommy W Farley, James Farmer, Kelly Farmer, Alex Fasciolo, Patricia Faulkner, Neil Fawcett, Heather Fedde, Taylor Feeley, Lisa Feichter, George Feil, Laura Feinstein, Dick Fejfar, Rae Fellenberg, Bret Feller, Sydney Feller, Cheryl Ferguson, Chris Ferguson, Jessica Ferguson, Meghan Ferguson, Robert Andrew Ferguson, Terri-Lynn Ferguson, Stephanie Fernandez, Mark Fiddelke,

 DOI : 10.1038/s41598-023-36928-1

James Fiedler, Madelyn Figueroa, Ruben Fijn, Tomis Filipovic, Debbi Fincher, Scott Finlayson, Michele Finn, Tom Finnegan, Dolores Fino, Gordon Firestein, Laurence Fischer, Frank Fish, Nick Fish, Amanda Fisher, Brian Fitzgerald, Deb Fitzgerald, Kelly Fitzgerald, Mariapaz Fitzgerald, Sue Fitzgerald, Mark Fitzmaurice, Steve Fiz, Maureen Flannery, David Fleetham, Charlotte Fleming, Colleen Fleming, Alana Flores, Ann Flower, Savannah Flowers, Mike Flunker, Michael Foegelle, Jackie Fogerty, Mike Fondiller, Richard P. Fontana, Bert Forbes, Amber Ford, Mareasa Ford, Becky Foresta, Reina Forsythe, John Y. Fortes, Tim Foster, Bob Fothergill, isaiah foulks, Amy Fowler, Jill Franceschini, Elia Franchini, Chelsea Francis, Kathy Francis, Matthew Franklin, Rose Franklin, Jason Franson, Sara Franson, Sonya Fraser, (Florence) Jeanette Frazier, Andrea Frederick, Jodi Frediani, Amy Free, Anne Freeman, Jonathan Freeman, Audra Freeze, Cj French, Evan Frey, Dana Friedman, Jeff Friedman, Dale Frink, Robin Froman, Rick Frontz, Janet Frost, David Fry, Jessica Fry, Carl Fucinari, Lou Fucito, Ritsuko Fujimori, Allison Fuller, Sara Furey, Bonnie Furman, Todd Furr, Tanya Gabriel, Jean-Denis Gagnon, Paul Gaillard, jenifer gaitan, Melissa Galieti, Joe Galkowski, Cara Gallagher, Robert D. Gallagher, Christopher Gallello, Sandra Galovičová, Sandeep Gandhi MD, Alisa Ganin, Karen Gannon, Tim Gannon, Patricia B Gans, Elizabeth Garcia, Erin Garcia, Martin Garcia, Eduardo García Aguilera, Artyn Gardner, Celia Garland, Colin Garland, Gemina Garland-Lewis, Diana Garrett, Jacob Garvelink, Anthony Garza, Sara Gasca, Oana Claudia Gasit, Gordon Gates, Kelly Gates, Rob Gaut, Roberto GDLV, Bob Geahlen, Rachel Gearhart, Charlie Geer, Joy Geeraerts, JD Geiger, Don Gelo, Chris Gentry, Gregg Gentry, Daniel Genuth, Rich German, Shane Gero, Diana Gerrans, Wayne Gerth, Benjamin Keith Getter, Richard Ghorbal, Brian Gibbs, David Gibbs, Donn Gibbs, Jeff Gibbs, Melissa Gibbs, Deborah Gibson, Linda Gibson, Winton Gibson, Maggie Gierard, Steve Gierke, John Gill, Verena Gill, Sierra Gilmore, Mark Girardeau, Glacier Wind Charters, Trista Gladstone, Petra Glardon, Don Glasco, Ronelle Glaza, Dennis Gleason, Deana Glenz, Erin Gless, Taylor Gliesman, Rhonda Glisson, Stephanie Go, Terry Godfrey, Ronan Goeke, Marina Gogl, Shiree Goins, Morgan Goloway, David Gomez, Steve Gompertz, Lynn Gong, Cesar Gonzalez, Marisel Gonzalez, Ursula Gonzalez Peral, Lauren Gooch, Sierra Goodman, Beth Goodwin, Jay Goodwin, Talia Goodyear, Debbie Goodykoontz, Madi Gordanier, Tom Gordon, Rosie Gordon Lennox, Tony Goring, Robin Gorsline, June Gothberg, Jonathan H Gottlieb, Doug Gould, Tina Gourlay, Glen Govier, Abril GR, Shannon Graber, Nick Graham, Tim Grams, Daniele Grandi, Frankie Grant, Lorraine Grant, Samira Grass, Kirstin Graves, Tim Gravis, Josh Gravley, Jackie Gray, Nicholas Gray, Lauren Graybill, Colin Greeley, Jacque Green, Kim Green, Melissa Green, Mike Greenfelder, Jim Greenough, Gina Marie Greer, Bob Greil, James Gresham, Keith Gress, Miriam Gribin, Janel Griffin, Leslie Griffin, Ellena Griffiths, David Grimes, Camille Grimshaw, Dave Grimshaw, Leo Groeneveld, Kassidy Groeper, Pia Gronvaldt, Tucker Grose, Hadley Gross, Mel Groulx, Debra Gruda, Frances Grunder, Keith Grundy, Janna Guay, Dan Gubitz, Selina Guckenbiehl, Torbjörn Gudinge, John Guilbault, Stephanie Guillot, Grace Guiney, John Gulleson, Aimee Gunzenhauser, Dianbo Guo, Roopali Gupta, Laura Gurak, Alexander Gustaveson, Roger Gutierrez, Janelle Guzman, Yvette Gwin, Jake Haber, Terry Haber, Isaak Haberman, Mary and Lou Haddad, Ian Hadgkiss, David Haeckel, Kevin Haehl, Stefan Hafke, Ben Hagler, Siri Hakala, Geoff Hall, Ian Hall, Kara Hall, Kevin Hall, Martin Hall, David Hallett, Richard Hallick, Grace Halliday, Michelline Halliday, Chris Halsch, Lynn Halsted, Bill Hamilton, Jarod Hamilton, Jeanne Hamilton, Lisa Hamilton, Loyd Hamilton, Richard Hamm, Joel Hammerman, Tim Hammond, Carol Hampton, Roy Hampton, Bill Handler, Dave Hanna, G Hannas, Cindy Hansen, Debbie Hansen, Holly Hansen, Jeff Hansen, Jeremy Hansen, Riley Hansen, Greg Hantak, Rick Harbo, Harbor Breeze Cruises, Venita Hardie, Misty Harding, DJ Hardy, Mark Hargrave, J Harles, Janine Harles, Conor Harlow, Jennifer Harper, Lauren Harper, Andrew Harpster, Elisabeth Harrell, Mark Harriman, Amanda Harrington, Dustin Harris, Joe Harris, Mark Harris, Summer Harris, Tammy Sue Harris, Kelly Harrison, Kathryn Hart, Randy Hartford, Christian Harvey, Dan Harville, Mark Haseltine, Cyndi Hash, Christina Hasiotis, Denis Haskin, Scott Hassler, Sarah Hauser, Terry Haven, Imagery Hawaii, Hawaiian Adventures Kona, Alayna Hawkins, Brian Hawkins, Marjorie Hawkins, Nikki Hawthorn, Rachelle Hayden, Susan Haydon, Floyd Hayes, Karen Hayes, Scott Hayes, Henry Hayhurst-France, Lee Hazelton, Carly Healy, Jamie Heater, Jackson Heather, Jerin Hedden, Heather Heffernan, Louisa Hege, Jill Hein, Stephen Heinz, Mike Hekkers, Kenneth Held, Rachel Helfing, Chris Helmore, Nancy Hemmelgarn, Dawn Henderson, Kyle Henderson, Brittany Hendricks, John Hendry, Garry Henkel, Brooke Henry, Thomas Hensel, Michael Hensley, Harvey Hergett, pam hergett, Maria Herrera, Teri Herrick, Kerrie Hesketh, Loriannah Hespe, Gudrun Heute-Bluhm, Nico Heyning, Paul Hibbard, Kevin Hickman, Chris Hill, Denise Hill, Diana Hill, Graham Hill, Jeff Hill, Marie Hill, Tim Hill, Cathy Hilliard, Megan Hillyer, Ray Hinojosa, Barbara Giner Hintermayer, Sharon Hirsch, Bert Ho, Amy Hobeika, Dana Hodde, Amanda Hodge, Tina Hoff, Michael Hoffhines, Patti Hoffman, John Hoffmann, Justin Hofman, Monica Hofmann, Simon Hogg, jeff hokanson, Anne Holder, Beth Holiman, Jack Hollands, John Hollerud, Damon Holley, Andy Hollingsworth, Mark Holm, Eric Holmes, Rena Holmes, Wil Holmes-Roys, Don Holmgren, Tom Holub, Tabatha Hommel, Charles Hood, Kate Hookham, Deb Hooyman, Alexia Hopkins, Ralph Lee Hopkins, Robert Horne, Era

Horton, Eric G Horvath, Kathryn Horvath, Lynn Horvath, Suann Hosie, Greg Hostetter, Amy Houghton, Karen House, Steve House, Kathleen Housel, Gerrit Houtschild, April Houweling, Alan Hovis, Mike & Anne Howard, Steven Howe, Megan Howson, Yui Hsieh, Feipeng Huang, Jean Huber, Ronald Huber, Stephen Huckvale, Julia Huddleson, Lauren Huddleston, Ian Hudson, Michael Huete, Kelly Hug, John Hughes, Lea Hughes, Jack Huisingh, José Humberto Hurtado Gutiérrez, Bryan Hunt, Walter Hunt, Steve Hunter, Tim Huntington, MS Roald Amundsen, Dave Huss, Mindy Huston, Royce Hutain, Stan Hutchings, James F C Hyde IV, Susan Iannucci, Donna Ikenberry, Sushanth Illuri, Sienna Imrie, Mike Ince, Breanna Ingwerson, Lynnette Innocent, David S Irland, Robert H. Irvine, Carol Irwin, Darren Irwin, Jessica Isaacs, Island Adventures Whale Watching, Islas Secas Resort, Nicole Ison, Candi Ives, Amanda Jackson, Chris Jackson, Dana Jackson, Larry L Jackson, Linn Jackson, Matt Jackson, Michael Jackson, Richard Jackson, Madison Jacobs, Lindsay Jaeger, Jay Jaffee, Sreekumar Jairaj, Don James, Heidi James, Kev James, Vanessa James, Roxann Janes, Terry Janes, Iyari Janethzy Espinoza Rodríguez, David Janka, Janet Janssen, Alvaro Jaramillo, Chip Jarvis, Joanne Jarzobski, Francisco Javier Ledezma López, Anvay Jawadekar, Rathish Jayabharathi, Tyler Jayy, Laura Jean Moreno, Christie Jeffers, Tim Jeffers, Sara Jefferson, Heather Jeffrey, Kimberly Jeffries, Andrea Jelaska, Connie Jellison, Carol Jenkins, Ed Jenkins, David Jensen, elaine jensen, Jennifer Jensen, Sarah Jensen, Trevor Jensen, Tye Jeske, Vince Jia, Ken Jinks, Richard Jioras, Donald Joeckel, Keni John, Ranger John, Jim Johns, Roy Johnso, Andrew Johnson, Ashley Johnson, Billy Johnson, Chris Johnson, Curtis Johnson, Greg Johnson, Jana Johnson, K Johnson, Michael Johnson, Paul Johnson, Robert E Johnson, Ryan Johnson, Sandra Johnson, Shelley Johnson, Todd Johnson, Tory Johnson, Tracy K Johnson, Don Johnston, James Johnston, Joe Johnston, Steve Johnston, Claude Joiris, Sandy Jolley, Clément Joly, Marion Joncheres, Angela Jones, Chris Jones, Jo Lynne Jones, Kayla Jones, Lindsay Jones, Mark Jones, Meagan Jones, Ryan Jones, Steve Jones, Marie Jorgensen, Amy Joseph, Jeff Jossendal, Mary Judy, Juneau Flukes Citizen Science, Kelsey Junkert, Mike Justa, Adam R. Kabir, Makaya kaduce, Heather Kahai, Marjorie Kaho'okele, Urmas Kaldveer, Tory Kallman, Arthur Kallos, Briana Kammer, Gerald W Kamprath, Cheri Kanaan, Russell Kanhai, Paul Kanive, Avinash Kar, Gevorg Karapetyan, Skye Karjian, Ben Karp, Kristen Kartisek, Jouko A Karvinen, Lubomir Kastovsky, Jaime Kasztelan, Ed Katrusik, Ally Kay, Diane Kayser, Jason Kazuta, Brendan Keane, Kiley Keatts, Chuck Keding, Nina Kee, scott Keefe, Ashley Keegan, Lotti Keenan, Sarah Keenan, Bill Keener, Peggy Keep, Janel Keim, Carol Keiper, Steven Keith, Jay Kellam, Vicki Kellar, Andrew Keller, Nathan Kelley, Whitney Kelley, Cristin Kelly, Declan Kelly, Mike Kelly, Robin Kelly, Sandy Kelly, Scott Kelman, Deanna Kelsey, Martin Kemper, Kimberly Kempfer, Andrew M. Kenefick, Dan Kener, Teng Keng Vang, Noga Kenigsztein, Faye Kennedy, Melinda Kennedy, Mike Kennedy, Patricia or Jim Kenney, Katelin Kenny, Hannah Kenzie, Wannes Kern, Geoff Kerr, Kaitlin Kerrigan, Natalie Kervin, Andrew Kessler, Wayne Kevern, Christa Key, Daniel Key, Colin Khan, Shaeesta Khan, Vivek Khanzode, Samantha Kidd, Wayne Kidder, Paul Killian, Tera Killip, Tom Kilroy, Donny Kim, EoJin Kim, Bobbi King, Jacob King, Miles King, Nicholas King, Ray King, Kaylie Kingsley, Kenzo Kiren, Sherry Kirkvold, Sara Kirlin, Jennifer-Justine Kirsch, Tammy Kirwan, Dmitrii Kiselev, Jenny Kish, Christine Kitchen, Christin Kless, Keith A Kline, Jamie Knaub, Marieke Knierim, Lucy Knight, Dayne Knowles, Gary A Knowles, Jens Koblitz, Capt. Mark Kocina of Avila Beach Whale Watching, Ryan Koehler, Jodi Koepke, Joshua Koester, Sue Koll, Deborah Komatsu, Julie Kondor, Dino Kondos, Emily Konopka, Sue Kopecky, Stacy Koppenhaver, Brandi Korte, Mark Kotcher, Matt Kouba, Emily Kounlavong, Gail Koza, Susan Kozdon, Dorota Kozub, Julie Krajewski, Trish L. Kramer, Sven Kranz, Caron Krauch, Paula Krauter, Amie Krebill, Madelaine Krehm, Rick Krejci, Blake Kremer, Andrea Kreuzhage, Andrea Krieser, Ryan Kroschel, alex krowiak, Alicia Krueger, Jamie Kruger, Basia Kruszewska, Anand P Kruttiventi, Jos Krynen, Diana Kuklok, Chetan Kulkarni, Harshal Kulkarni, Jessica Kunkel, Irene Kurata, Libby Kurt, Jonathan Kush, Emma Kuske, Daniel Kyba, Jennifer Lacavera, Julie Laderach, Jody Ladwig, Chellie LaFayette, Desmond Lai, Hamish Laird, Susan Lambert, Neil Lamka, Amelia Lamonde, Andre Lamouche, Rachel Lancaster, P Lander, Paul Lander, Mimi Landers, Mike Landis, Bekah Lane, Sheryl Lane, Tamara Lang, Ski Laniewicz, Lisa LaPointe, Nathaniel LaPointe, Timothy Lara, Maureen Lare, Nicole LaRoche, Nathan Larson, Greg W. Lasley, David Lates, Morgane Lauf, andrea Laughlin, Nicole Laureys, Christopher Laurinec, Rasmus Laursen, Kristien Lauwers, Jeremy Lavender, Ryan Lawler, David Lawrence, Jennie Lawrence, Robert Laycock, Beth Layman, Pamela Le Noury, Aurora Leal, Allen Leblanc, Gary Lebsack, Manuel Ledesma, Annabelle Lee, Brian Lee, Ed Lee, Ingmar Lee, Isabel Lee, Olivia Lee, Yousup Lee, Mike Lees, Mark Lefkin, David Leftwich, Capt. Alexander Legaspi, Tasha Lehman, Mary Leigh Henneberry, Rob Lejsek, Bud Lensing, Leo Leon, John Leonard, Kathleen Leonard, Melissa Leonard, Randy Leonard, David Leong, Captain Tara Leota, Jordan Lerma, Sara Lesser, Pam Lever, Josh Levinger, Susan Levinson, Kristina Lewandowski, George Lewbel, Dylan Lewis, Jim Lewis, Joni Lewis, Kamla Lewis, Linda Lewis, Marty and Carol Lewis, Teresa Leyva, Yangjie Li, jennifer libotte, Brad lidstone, Maureen Lien, Kurt Liewer, Shannon Lightfoot, Allan Ligon, Rachel Lilly, Karac Lindsay, Yifan Ling, Bob Lippert, Albert Lipson, Jeffrey Liroff, Kevin Lisota,

Nicole Litkovitz, Ann Littlefield, Jeff Litton, Leah Liu, Victor Liu, Live Satellite News, Patti Llacsa, Bridgett Locken, Doug Lockhart, Thom Loeffler, Holly Lohuis, Daniela Lomelin, Brittany Long, Carter Long, Gabrille Lopez, Laura Lopez, Tony Lopez, Ciro Manuel Lopez Leal, François Lord, Randy D Lord, Samuel Lott, Betina Loudermilk, Angela Lovell, Robin Lowe, Stephen Lowe, Andrew Lucas, Isabelle Lucas, Lea Lucas, Deborah Lucia, Barb Luck, Angelika Ludwiczak, Cara Lueders, Eduardo Lugo Cabrera, Jared Luther, Seth Luther, Bill Lutz, Marty Lycan, Shelley Lynch, Janice Lynn Nath, Ruth MacGregor, Heather MacIntyre, Lilli Mack, Sally Mack, Donald L MacKinnon, Julie MacKinnon, Rory Macklin, Jeffrey MacQuarrie, Steven Madow, William Maes, Kaitlin Magliano, Megan Magnant, Gerry Mahoney, Karen Mahoney, Diane Mahovlic, Adam Maire, Shea Majbroda, Chris Makowski, K Makowski, Daniela Maldini, Rebecca Malkewicz, Michael Malone, Rebecca Malone, Shannon Malone, Lars Maltha Rasmussen, Divya Mankikar, Calvin Mann, Tina Mann, Cynthia Manning, Michaela Mansholt, James Manzanares, Erin Marchand, Guylaine Marchand, Mary Marchetti, Leon Marciak, Maria Laura Marcias, Michelle Marckwardt, Aurelie Marie, Isabela Marino, Amy Markowich, Tim Markowitz, Jennifer Marks, Stephanie Marks, Melody Marler, Eva Marley, Cindy Marple, Randy Marsh, Rhys Marsh, Stephen Marsh, Eric Marshall, Skylar Marshall, Hella Martens, Clive Martin, Don Martin, Joel Martin, Tim Martin, Jacob Martinez, Melissa Martinez, Cynthia Martinez Hernandez, Tyler Masden, Kate Mason, Sloan Massie, Kera Mathes, Chelsea Mathieson, Aditi Mathur, Howard Matis, Betty Matsushita, Lesley Matthews, Jill Matyuch, James Maughn, Gale Maxey, Colin May, Laurence May, James Maya, Bijan Mayelzadeh, Chelsea Mayer, Jane Mayer, Tonia Mayerle, Jennifer Mayhan, Anna Mazurek, Lori Mazzuca, Frank Mc Cann, Peter B McAllister, Clay McBride, Tim McBride, Colin McCann, Tiffany McCann, Claire Mccarty, Lauren McCaslin, Julie McClain, Forrest McClellan, Barbara Mcclure, Bill McComb, Matt McCombs, Jake McConnell, Shawn McConnell, Terry McConnell, Molly McCorkell, Greg McCormack, Alex McCormick, Michelle McCune, DVM, Dane McDermott, Katherine McDermott, Marissa McDermott, Neil McDermott, Bryan McDonald, Davis McDonnell, Kathee McFarland, Mike McGee, Jamie McGourlay, Owen Mcgourlay, Olivia McGovern, Murray McGregor, Tim McGue, Michael McGuire, Cristin McKee, Andrea McKenney, Deanne Mckenzie, Ella McKhann, Brooke McKinley, Peggy Mcknight, Laura McLain, Janet McLaughlin, Don McLeish, Keith McMahan, Raja McMahon, Rebecca McManama, Ian McNair, Sharlene Mcneish, Liah McPherson, Dan McSweeney, Toni Meador, Deanna Meadows, Victoria Medina, Luis Medrano, Pam Meek, John Meier, Dorothy Meinhold, Natan Melamed, Walter Enrique Melendez Herrera, Brandon Meligan, Beth Mellas, Matt Mellendorf, Brett Mello, Sonia Mellorr, Juan Mena, Frank Mendel, Rodrigo Menezes, Susan Menton, Gerard Menut, Gersld Mercier, James Meredith, Rachael Merrett, Nancy Mesa, Valerie B Messier, Leticia Metherell, Matt Metzger, Emily Meucci, Joe Meuleman, Jessie Meyer, Rob Meyer, Joshua Meza-Fidalgo, Celine Midavaine, Dan Midea, Susan Mikolatcher, Elanor Miller, McKenna Miller, Randall Miller, Sheila Miller, Wendy Miller, Sally Milligan-Smith, Jason Mills, Michelle Mills, Sydney Minges, Alan Mirly, Anika Miskar, Shelby Miskell, Allie Mitchell, Baleigh Mitchell, Eric Mitchell, Jessica mitchell, Carla Mitroff, Tomoko Mitsuya, Matt Mittermayer, Joe Mobley, Mario Modena, Levonne Moise, Alex Molina, Annette Moll, Steven Moll, Paz Molnar, Kari Monagle, Peter Monson, Monterey Bay Whale Watch, Karen Montero, Megan Montes, Maddy Montgomery, Mark Montgomery, Sara Montour Lewis, Cynthia Moody, Scott Moody, Stacy Moody, Charles Moon, Andrew Moore, Barrie Moore, Christine Moore, Ginger Moore, Jimmy Moore, Lorellen Moore, Shannon Moore, Slater T Moore, Terri Moore, Alison Moors, Juan Morales, Marvin Morán, J Moreau, Heather Morgan, Kat Morgan, Lee Morgan, Linda Morgan, Patricia Morine, Ann Morris, Ashley Morris, Kristin Morris, Noelle Morris, Roger Morris, Breck Morrison, Christa Morrison, Ryan Morrison, Stacey Morrison, Culley Morrow, Bonnie Morup, Karen Moses, James Moskito, Cyndi Moss, Michael Moss, Tressa Mower, Tammy Muckway, Paul Mudgett, Robert Mueller, Clemens Muenzer, Sonja Mugg, Maureen Mullen, Damon Mullins, Kristine Mulloy, Jeff Muncy, Peggy Mundy, Theresa Muray, Angela Murphy, Daniel Murphy, Karyn Murphy, Pat Murphy, Russell Murphy, Sam Murphy, Laura Murray, Nick Murray, Jared Musgrove, Mark Mushkat, Jeff Muskopf, Tom Mustill, Karen Myers, Mark Myers, Renee Myers, Sandy Myers, Sheila Myers, Tom Myers, Rob Myles, Mollie Naccarato, Pete Naik, Raghavan Nair, Emily Nalbach, Gabriella Napier, Graham Napier, Calvin Nash, James Naughton, Kay Naumann, Nautilus Whale Watch, Carlos Navarro, Gabriel Navarro, Ryan Navazio, Lena Nazarek, Kerry Nechodom, Sean Neilson, Rick Neipert, Emma Nelson, Kyle Nelson, Michael Nelson, Sarah-Mae Nelson, Kimberly Nesbitt, Neil Nesheim, Al Ness, Matthew Ness, Mollie Ness, Dan Neumann, Gary Neumann, Kimberly New, Ken Newcombe, Carrie Newell, Hayley Newell, Will Newhouse, Stephanie Newkirk, Erica Newland, Chris Newsom, Kathy Newstead, CK Newton, Richard Newton, Savanna Nice, Linda Nichol, Brian Nichols, Gary Nichols, Krystal Nichols, Sandra Nichols, Lorain Nicholson, John Nickel, Caitlyn Nieblas, Ron Niebrugge, Brian Niedbalski, Jill Niederberger, Jill Niederstadt, Jodi Nieding, Brie Nielsen, Lisa Nielson, Katie Niesen Cook, Nirupam Nigam, Katie Nightengale, Huub Nij Bijvank, Alan Niles, Sarah Nissen, Allen Nix, Mike Nolan, Mary E Nolen, Kathy Nolet, Michael

 DOI : 10.1038/s41598-023-36928-1

Noonan, Leif Nordman, Meredith Nordmann, Lyndsey Norman, North Shore Sailing Adventures (Keet), Chuck Northcutt, Mark Northwood, Blaine Norton, Mairead Norton, Paul Norwood, Christian Nova Juarez, Karla Nyquist, Kenneth O'Brien, Pat O'Flaherty, Liza O'Hare, Julia O'Hern, Becky O'Neill, Bruce O'Leary, John Oates, Mark OBrien, Tjasa Obrulek, Cornelia Oedekoven, Offshore Blue Adventures, DJ Ogden, Alison Ogilvie, Kurt Ohlander, Haruna Okabe, Doug Oldham, Mike Olds, Jeff Oliver, Mary Oliver, Rich Olmstead, Lars Olofsson, Carrie Olson, Terry Olson, Cheryl Opfer, Steve Orchard, Sara Orear, Denny Organ, Olga Organvidez, Tammy Orr, Rachel Orrell, Carrie Orsmond, Andre Ortega, Ruben Ortiz, Uma Orvañanos, Dakota Osborne, Emily Osborne, David Osherow, Maren Oslac, Julie Oswald, Steve OToole, Out to Sea Expedition Company, Todd Owens, Peter Oxman, Karlee Oyama, Iacopo Pace, Ralph Pace, Erica Page, Kaitlyn Page, Patty Pai, Judy Painter, Sheryl Painter, Katharina Palffy-Gelfand, Stephen Palmer, Steven Palmer, Anurag Pande, Warren Panem, Maggie Pang, Amy Pantoya, Marjorie Paoletti, Paul Papanek, Amalia Parecki, Kate Parker, Michele Parker, Susan Parker, Steve Parkin, Lynne Parkinson, Jim Parks, Sunil Parmar, Robert Parsons, Arpana Parti, Chanel Partlow, Drew Pasek, Paso Pacifico Nicaragua, Elisa Passarelli, Maygan Patch, J. R. Patee, Aarya Patel, Katie Patrick, Shaun Patrick, Travis Patten, Caroline Patterson, TJ Patterson, Judy Patterson Ayala, Debbie Patton, Robin Pavey, Allison Payne, Lucy Payne, Andrew Peacock, Donna Peake, Chris Peal, Johanne Peale, Jules Pearson, Meghan Pearson, WC Pearson, Brandon Pease, Kathy Peavey, Don Pedersen, Senta Peirsol, Kate Pennington, George Penokie, Cathy Pepperd, Don Pepperell, Vanessa Percival, Angel Perdomo, Sarah Perdue, Andrew Pereira, Anthony Perella, Faviola Perez, Rene Perez, Carlos Enrique Pérez, Manuel Pérez Castillo, Sue Perin, Herb Dan Perry, Kate Perry, Kevin Perry, Robert Perry, Cheryl Peteherych, Phyllis Peter, Cheryl Peters, Paul Peters, Aaron Peterson, Donna Peterson, Jeffrey Peterson, Dean Petterson, Brianna Pettie, Judith Pettit, Sherry Pettite, Jilly Pfifferling, Jim Pfitzer, Cheryl Phalen, Frederick Phaneuf, Britt Philbin, Jeff Phillippe, David Phillips, Tarah Phillips, Traci Phillips, Kim Phillips-Francis, Bob Phreaner, Enid Phreaner, Kathryn Picardo, Simon Pidcock, T. Pieper, Janah Pierce, Mary Pierce, Meredith Pierce, Michael Pierson, Pablo Piña, Amy Pingree, Rebeka Pirker, Ann Pitts, Bob Pitts, Dean Pittsinger, Diane Pizza, Nicole Pizzini, Bruce Plackowski, Joseph Podell, Ally Pohl, Colleen Poland, Katherine Pollock, Donna Pomeroy, Thomas Pons, Linda Ponter, Tiffany Poon, Andrea Popick, Edward D Porter, Marshall Porter, Nicene Porter-Shimotomai, Susan Portnoy, Mike Portt, Nastasia Poso, Cameron Powell, Kim Powell, Ray Powell, Steven Powell, Kristin Powers, Adele Prandini, Bev Prefontaine, Elizabeth Premo, Andrew Prestwich, Andy Price, Brennan Price, David Price, Nathan Primus, Scot Prince, Steph & Oli Prince, Courtney Prinke, James Pritchard, Marleen Probert, Rob Provost, Annick Pruett, Paul Pudwell, Scott Pudwell, Elfyn Pugh, Earl Pullen, Alecia Pulver, Douglas Punzel, Proyecto Pupila, Erin Purdy, Jill K Quaintance, Lucy Quayle, Whale Quest, Robert Quiggle, Morgan Quimby, Adam Quinn, Neal Quinn, Norman Quinn, Magaly Quintana, Dale Raaen, Laurie Racenet, Carolyn Radlinger, Rachelle Radpour, Linda Raffray, Damian Rafie, Genevieve Rafie, Anisha Raghavan, Shulynn Ragland, Christopher Rahayel, Maggie Rahm, Robert Raimo, Logan Rainey, Theresa Rainey, Abhishek Rajbhandari, Bud Ralston, Andrea Rambeau, Morgan Ramirez, Kimberly Ramos, Christian Ramp, Catherine Ramsey, Louis Rana, Kimberly Randal, Patrick Randall, Scott Ranger, Shannon Rankin, Sarah Ranney, Ken Rapp, Leslie Rapp, Shannon Rapuano, Aisha Rashid, Kreig Rasmussen, Dana Ratliff Howson, Kristina Rau, Laura Rauscher, John Raven, Aaron Raymond, Mohammad Raza, Katie Read, Laurie Recchio, Morgan Rector, Adrian Redd, Taylor Redmond, Dave Redstone, Alyssa Reed, Mike Reed, Christine Regent West, Cindy Reich, Terrie Reichling, Clifford Reid, Rich Reid, Robert Reid, Joy Reidenberg, Rhonda Reidy, Petra Reiman, Kelsey Reimer, Nona Reimer, Dennis Rein, Ashley Rendon, Martin Renner, Michael Reppy, Paul Resch, Heather Reser, Connie Respicio, Sven Reutter, Vinay Revandkar, Marc Reynolds, Matt Rhoad, Justin Rhodes, Selena Rhodes Scofield, Curt Ribando, Tagnan Ribaudo, Hannah Rich, Janis Rich-Smith, Brent Richardson, Chris Richardson, Gail Richardson, James Richardson, Joanne Richardson, Michael Richlen, Nicky Richman, Leslie Richter, Jonathan Rigby, David Rigden, Brandy Riggan, Jade Riggins, Shawn Riggins, Robyn Riley, Sunomen Rimel, Stephen Rink, Rebecca Ripp, Bert Risseeuw, Thomas Ritchie, Shannon Rivas, Angela Rivera-Nieves, Clint Rivers, Theresa Rizza, Jessica Roame, Capt Nicholas Robbins, Holly Robbins, Emily Roberson, Frank Roberts, Jeremy Roberts, Katie Roberts, Lindsey Roberts, Melissa Roberts, Tamara Roberts, Christy Roberts-Clark, Diana Robertson, Chloe Robinson, Everett Robinson, Jim Robinson, Nikki Robinson, Christopher Robson, Matt Robson, Cindy Rocha, Jeff Rockwell, Rachel Rodell, Rosmely Rodríguez, Benjamin Rodriguez, Bryan & Tammy Rodriguez, Jill Roehl, Jack Roesch, Dave Rogers, Dennis Rogers, Rebecca Rogers, Steve Rogers, Pierre Röglin, Nicole Rojas, Shana Rollins, Rocio Roman, Carmen Romano, Mike Romano, Peggy Romfh, Fernando Romo, Taylor Ronan, Valerie Rondone, Dennis Roode, Edward Rooks, Katie Rooks, Rosie Roppel, Benjamin Rose, Hugh Rose, Ed Roseboom, Don Roseborough, Nina Rosen, Robert Lynn Rosenthal, Scott Rosman, Amy Ross, Kristen Rosselli, Mike Rost, Jon Roswurm, Lori & Rich Rothstein, Frank Rotter, Lisa Roudabush, Yves Roumazeilles, Donna Rowe, Michelle Rowlands, Rachel Rozin, Cit Rubio, Jane Rudebusch,

 DOI : 10.1038/s41598-023-36928-1

Rand Rudland, Dominik Ruegsegger, Jane Ruffin, Ashley Ruis, Cecilia Ruiz, Dean Rummel, Eloise Russell, Jayson Ruth, Gina Ruttle, April Ryan, Jaime Ryan, Sara Ryan, Sarah Ryan, Kristi Ryken, Brad Ryon, Kiera Ryon, Richard Ryon, Sandy Sabados, Erica Sackrison, Kayla Saco, Matt Sadowski, Kyndace Safa, Karlee Sager-Christenson, Jasspreet Sahib, alexander Sakharov, karen saklad, Eva Salitus, Keith Salmon, Sabrina Salome, Andrei Samofalov, B Sanchez, Paulina Sánchez, Anna Sanchez Mora, Monica Sandberg, Lindsay Sandbothe, Lori Sandercock, Eric Sanders, Shayne Sanders, Stephen Sanders, Marybeth Sanders-Wilson, Luis Jesús Sandoval Hernández, Daniel Sands, Shannon Sands, Adria Sandusky, Jerry Sanger, Santa Cruz Whale Watching, Gabriel Santana, Zaahir Santhanam, Laura Santillan, Selena Santos Rivera, Holly Sargeant-Green, Yoko Sathorn, Haruko Sato, Tom Satre, Claire Savage, Megan Savage, Timothy Savage, Tom Savage, Bill Savory, Rick Sawicki, Elizabeth Sawyer, Mark Sawyer, Al Saxon, Molly Saxton, Ginny Sayre, Tracey Scanlon, Jay Scarlett, Dave Schaar, William Schaefer, Bob Schafer, L Schallop, Amanda Schaub, Andy Scheffler, Ashley Schenkel, Renee Schlichting, Jessica Schloemer, Amber Schmalz, Derek Schmidt, Brian Schmitt, Barbara Schmitz, Paul Schofield, Pamela Scholten, Dustin Schoonveld, Mark Schraad, Mary Schrader, Nicole Schriber, Keith Schroder, Russell Schuetz, Alicia Schuler, Terry Schuller, Ken Schultz, Peter Schulze, Alli Schumacher, Steffen Schwab, Dana Schwartz, Christian Schwarz, Beth Schweiner, Linda Schweitzer, Remo Sciessere, Karla Sconiers, Steve Scordino, Michael Scott, Skipper Scott, Sarah Scrivano, Sea Safari Huatulco, Lisa W Seaborn, Christina Seals, Ken Seddon, Trinitie Seeber, Gretchen Sell-Finley, Lisa Senitte, Chris Senopoulos, Amy Serafino, Cheryl Serle, Gabriel Serrano, Kenneth Setter, Lauren Seuch, Scott Severn, Suzanne Sevigny, Frank Sew Atjon, Dawn Seymour, Gitesh Shah, Malhar Shah, Mehul Shah, Pavak Shah, Vaishali Shah, Linda Shaka, Michael Shane Long, Jessica Sharman, Susan Sharon, Holly Sharp, Bill Sharpes, Alexander Shaw, Vincent Shay, Glenn Sheckells, Jessica Shefferd, Gabriel Shekleton, Kim Shelden, William Shelden, Kim Sheldon, Paul Sheldon, Tom Shelley, Jason Shellock, Jerry Shenk, Brian Shepard, Katherine Shepherd, Steve Shepp, Steve and Alison Shepperson, Bryanna Sherbo, Skylar Sherbrooke, Steven Sheridan, Bethany Shimasaki, Sara Shimazu, Tomoko Shimotomai, Ken Shirriff, Delaney Shoemaker, Valerie Shore, Emma Shrack, Michael Shron, Benjamin Shulman, Fawn Shupp, Lisa Sichi, Elicia Sick, Muzaffar Siddique, Alex Siddons, Nils Siefen, Paige Siegel, Kátia Silva, Eden Silver, Beverly Simmon, Frank Simms, Amber Simon, Megan Simons, Edith Simonson, Debby Simpson, John Sims, Tai Sing Hee, Amy Sirota, Donna Skiba, Alexia Skrbic, Raven Sky, Deborah Slavin, Todd Slawson, Loretta Sloan, Gregory Slobirdr Smith, Scott Slone, Nathan Small, Allison Smith, Angela Smith, Annette G.E. Smith, Blake Smith, cari a smith, Carissa Smith, Darin Smith, Darla Smith, Ed Smith, Elizabeth Anne Smith, Grady Smith, Hannah Smith, Jamie M Smith, Jason smith, Jeff Smith, Jill Smith, Maria Smith, Mark Smith, Monica Smith, Nichole Smith, Nichole Smith, Patty Smith, Paul Smith, Pete Smith, Ryan Smith, Sherrill Smith, Steve Smith, Wayne Smythe, Kendall Snel, Maggie Snelgrove, Taylor Snooks, Charles Snyder, Shawn Snyder, Susan Snyder, Thomas Snyder, Joanna Sokolowski, Gonzalo Solernou, Marty Solis, Adriana Solomon, Berit Solstad, Jeanne Sommet, Gabriela Somohano, Drew Sorensen, Anton Sorokin, Dawn Sorrell, Eric Spangler, Michelle Sparks, Paige Sparks, Tobin Sparling, Elizabeth Sparrow, Elizabeth Spear, Don Spencer, Kate Spencer, Kayla Spencer, Scott Spickler, David Spiegel, Anne Spiggle, R. Spooner, Jay Spring, Jack Springer, Matthias Springer, Patrick Sproul, Chris Spurgeon, Cherie St. Ours, Joel Stack, Lori Jean Stackhouse, Chris Stafford, Kate Stafford, Tom Stafford, Pamela L Stahlak, Anna Kate Stalker, Mike Stallard, Susan Stalzer, Cindy Stanfield, Mollie Stanfield, Amber Stanfill, Debbie Stanford, Alex Stanton, Wayne Staples, Valerie Stapleton, Tammy Starkey, Jennifer Starr, Erich Starzinger, Doug Stecklein, Michael Steele, Joyce Stefancic, William Stehl, Amy Steider, Sharon Steinbis, Valérie Stekke, Hazel Stephens, Laura Stephens, Karen Stepps, Jon Stern, Lisa Stern, Dan Sternau, Dionna Stewarr, Adam Stewart, Bill Stewart, Deb Stewart, Julie Stewart, Steven Stock, Richard Stockley, Jenna Stoddard, EJ Stokes, Isabelle Stoll, Joe Stoltz, Petra Stolz, Ananda Stone, Philip Stone, Thomas Stone, James C Straith, Randy Straka, Ryan Stramp, Carolyn Straub and Steve McHenry, Gene Streagle, Kurt Strueh, Sarah Strukan, Stubbs Island Charters, Bill Sturgeon, Edwin J Suarez, Nicole Sucre, Pamela Sufi, Tracie M. Sugo, Lawren Sullivan, Jen Summers, Paul Suppo, Mark Susol, Maria Sutherland, Ed Sutter, Willi Sutter, Debbie Sutton, Gary Sutton, Kim Sutton, Renita Swaim, Sarah Swain, Mark Sward, Jim Sweet, Steve Swen, Jennifer Swenson, Walter Swett, Abby Swisher, Phil Sykes, Patrick Sysiong, Monica Szczepanski, Elke Szelwis, Magdalena Szymanska, Jon Taets, Dorinda Talbot, Patricia Talley, Chere Tamura, Andrew Tan, Bonita Tanner, Patrick Tanner, Dario Taraborelli, Anay Tarnekar, Martin Tassche, Emily Tate, Robert Tatum, Deanna Taubman, Andrew Taylor, Barbara Taylor, Courtney Taylor, Greg Taylor, Katlyn Taylor, Lisa Taylor, Shelly Taylor, Wayne Taylor, Manibuka Teaeki, Jami Teal, Kristin Telfer, Jeff Tell, Jennifer Tenneson, Hayden Terjeson, Todd Ternovan, Richard Ternullo, Scott Terry, Marisa Tervoort, Bruce Terzes, Owen Tesson, Sam Test, Frank Tetreault, Sheevaun Thatcher, Carol Thaxton, Julia Thelemann, Cassie Thiel, Gloria Thoburn, Jared E Thoma, Cathryn Thomas, Charles A. Thomas, Dexter Thomas, Gary Thomas, Jason Thomas, Jeremy Thomas, Lucy Thomas, Tony Thomas, Debi

Thompkins-Neisius, Debbie Thompson, Michael Thompson, Tori Thompson, Jordy Thomson, Rob Thomson, Sam Thomson, Julia Thorman, Bryan Thorne, Sheila Thornton, Sharon Thorp, Rhianna L. Thurber, Tamara Thurmond, Makayla Tideman, David Tidgwell, Jennifer Tietz, Bob Tilden, Kristy Tillotson, Paul Timmis, Cees Tineke, Ming Ting, Hailee Tocco, Karen Tocque, Edward Todd, thomas tohill, Toody Tolley, Josie Tolliday, Kurt Tometich, Helen Torino, Amanda Torre, Kira Torre, Jared Towers, Ashton Josephine Towne, Tracy Townsend, Margo Tracey, Steven Tracey, Bryan Trammell, Tranquilo Surf Adventures, Cynthia K Trapanese, Alexa Trauger, Amy Tresenrider, Gregory Tressler, Alexander Trienen, Yubarta Trips, Gerard Troost, Yves Trottier, Delaney Trowbridge, Kristina Trowbridge, Declan Troy, Bill Truitt, Sheri Trupp, Charmaine Tsoi, Gerard Tubbing, Bill Tucker, William Tudor, Linda Tuero Lindsley, Phil Turcotte, Andy Turner, Jordan Turner, Kim Turner, Steve turner, Christy Turnlund, Ashala Tylor, Adam Ü, Marcy Ubois, Ken-ichi Ueda, Brian Ullestad, Barry Umstead, Erin Underwood, Amanda Urena, Fernanda Urrutia, Randy Uyhely, Charles Vadovic, Kelly Valdez, Kim Valenta, Tim Valentine, David Valerio, Susan Valerio, G Valley, Nate Vallier, Leendert van Bochoven, Mike Van Camp, Deborah van de Water, Daniel van der Brug, Rebecca van der Grient, Talitha van der Linden, Mylene van der Scheer, Alan Van Dyke, Christoff Van Hoecke, Marc van Ingen, Fabio van Roon, Robert Van Winkle, Trish VanBeek, Andrea Vance, Sophia Vancouver, Vancouver Island Whale Watch, Leah Vanderwiel, Sheri Vandrew, Elie Vannier, Patricia VanOver Indictor, Bradley Vanston, Norm Vargas, Jenny Varley, Molly Varner, Raquel Vasquez, William Vaughn, Marlenne Vazquez Cuevas, Gregor Veble Mikic, Scott Veirs, Michelle Velasco, Casey Vellutato, Amy Venema, Sriram Venkateswaran, Kirstie Venton, John Verhey, Thomas Vermeulen, Tina Vermeulen, Thomas Vermillion, Kiana Viceroy, Jeff Vidal, Ryan A. Vienna, Justin Viezbicke, Lorena viloria, Len Vincenti, John Vinci, Corey Vink, Warren Vinzant, vincent visser, Tim Vitoux, Carlos Vivares, Andrew Vlack, Jaap Voets, Mandy Volpe, Vincent von Kaenel, Oliver Vossen, John Vraspir, Jordan Wade, Kathy M Wade, Amanda Wadsworth, Emily Wagner, Matt Waibel, Doug Walker, Lisa Walker, Robin Walker, Walter Wall, Cara Wallschlaeger, Wally's Whales Tales, Gretchen Walsh, Wyatt Walsh, Traci Walter, Kylee Walterman, Mark Waltermire, Benton Walters, Lauren Walthour, Tracy Walton, Maris Ward, Sam Ward, Scott Ward, Sharon Wardale Trejo, Christy Ware, Robert Warner, Elizabeth Warren, Mike Warrender, Tom Warshawski, Michael Waterman, Rhys Watkin, Marley Watkins, Robert Watkins, Nicole Watson, David Watts, John Watts, Jana Weaver, Marc Webber, Megan M Weber, Tiffani Weber, Michelle Weidenbach, Elisabeth Weimar, Holly Weinstein, Cali Weise, Kim Weiss, Niki Weiss, Brenton T Weist, Carmen Welbon, June Weldon, Neal Wells, Jane Wembridge, Lars Wensel, Sena Werdegar, Charlotte Werts, Kathy West, Kylie West, Louise West, Matthew West, Tammy West, Peter West Carey, Christopher Westby, Bob Western, Emily Westman, Robert Whale, Whale Watch Cabo, Whale Watch Vallarta, Connor Whalen, Lisa Wheeler Foley, Thomas Whelan, Jack Wherley, Amy White, Chantal White, Eugene White, Michael White, Dee Whitehurst, Tanya Whitman, Jonathon Whitney, James Whittaker, Bruce Whittington, William Whobrey, Emily Whymark, Wendy Whyte, Patrick Wieler, Sigi Wiesner, Kelley Wiggins, Lynn Wild, Wild Side Specialty Tours, Kootenay Wilderness, Eric Wildt, Joan Wilensky, Darryl Wilkins, Steve Willetts, Clint William, Becky Williams, Blake Williams, Erik Williams, Ernest Williams, Les Williams, Melanie Williams, Miranda Williams, Paul Williams, Tom Williams, Barry Willis, Lucy Willis, Bobbi Wilmoth, Deon Wilner, Ashley Wilson, Betsy Wilson, Elijah Wilson, Elizabeth G. Wilson, Eric Wilson, Gary W Wilson, Jane Wilson, Jenifer Wilson, Jim Wilson, Kristi Wilson, Lili Wilson, Maria Wilson, Ryan Wilson, Scott Wilson, Frankie Wilton, David Wimpfheimer, Steven Wincor, Becca Winegar, Brenden Winters, David Winward, Erica Wirth, Sara Wise, Darlene Wisecup, Melissa Witek, Andrew Wittkower, Chris Witwer, Jackie Woenker, Andy Wolcott, Patrick Wolf, Gregory Wolfe, Erica Wolff, Ron Wolotzky, Edmond Wong, Mona Wong, Tom Wong, Claire Wood, Glen Wood, George Woodland, Cynthia Lynn Woodruff, Robyn Woods, Steve Woodward, Carl Woog, Karen Wooldridge, Kristina Wooldridge, Koren Woolner, Ross Wopat, Brady Worstell, Donna Wotton, Jack Woychowski, Janie Wray, Lynn Wright, Neysa Wright, Sandy Wright, Angie Wrightson, Trond Wuellner, Frank Wurtzel, Gerry Wynkoop, Kate Wynne, Lily Xie, Jingjing Xue, Alex Yale, Peter Yamamoto, Joey Yang, Emlyn Yoon-Buck, Carl Yoshihara, Kevin Yoshimoto, Bob Young, Chloe Young, Zangnan Yu, Yubarta Ecoturismo, Katie Zacarian, Hanane Zahrouni, Holly Zavaleta, Stefen Zeff, Bryan Zeitler, Liz Zele, John Zelezny, Faith Zelinsky, Yibo Zeng, Jeffrey Zens, Addison Zenthoefer, Kate Zenthoefer, Kyle Zentner, Karen Zepeda, Luis Pedro Zepeda Celis, Melissa Zeroth, Christina Zhang, Hanlin Zhang, Jiahe Zhang, Hongming Zheng, Ted Zhou, Brittany Ziegler, Matt Ziegler, Lory Zimmeman, Sandi Zimmermann, Ed Zimny, Ann Zoidis, Heather N Zoric, Matea Zosak, Erica Zubris, Roma Zuniga, Elizabeth Zwamborn, Zoe Zywiak, Diving Service Ashibee Blue (Yukiko Yamada), Okinawa Island Crew (Koji Yamamoto), Onna-son Whale Watching Association (Masaru Katayama), Reeffers Ocean Sports (Koji Sakazaki), Top Marine Staff, Marine House Seasir Staff, Total Marine Spot News staff, GTdivers (Kazutoshi Uehara), Gokurakucho (Taketomo Shiratori)
